# Quantification of electrochemically accessible iridium oxide surface area with mercury underpotential deposition

**DOI:** 10.1126/sciadv.adp8911

**Published:** 2024-11-06

**Authors:** Jane Edgington, Sejal Vispute, Ruihan Li, Adrien Deberghes, Linsey C. Seitz

**Affiliations:** Department of Chemical and Biological Engineering, Northwestern University, Evanston, IL 60208-3113, USA.

## Abstract

Research drives development of sustainable electrocatalytic technologies, but efforts are hindered by inconsistent reporting of advances in catalytic performance. Iridium-based oxide catalysts are widely studied for electrocatalytic technologies, particularly for the oxygen evolution reaction (OER) for proton exchange membrane water electrolysis, but insufficient techniques for quantifying electrochemically accessible iridium active sites impede accurate assessment of intrinsic activity improvements. We develop mercury underpotential deposition and stripping as a reversible electrochemical adsorption process to robustly quantify iridium sites and consistently normalize OER performance of benchmark IrO*_x_* electrodes to a single intrinsic activity curve, where other commonly used normalization methods cannot. Through rigorous deconvolution of mercury redox and reproportionation reactions, we extract net monolayer deposition and stripping of mercury on iridium sites throughout testing using a rotating ring disk electrode. This technique is a transformative method to standardize OER performance across a wide range of iridium-based materials and quantify electrochemical iridium active sites.

## Teaser

A robust electrochemical adsorption technique enables iridium active site quantification for catalyst activity normalization.

## INTRODUCTION

Hydrogen gas (H_2_) is a critical global chemical and fuel involved in key industrial processes, including the production of ammonia, which sustains the global population’s agricultural needs. However, the production of H_2_ currently relies on carbon-intensive steam reformation processes that, on average, emit 10 metric tons of CO_2_ per ton of H_2_ created (*1*, *2*). Alternatively, proton exchange membrane water electrolysis (PEMWE), when using renewably produced electricity, is a carbon-free process of H_2_ production. As the cost of renewably produced electricity plummets and the demand for carbon-free technologies grows, PEMWE becomes an increasingly promising and sought-after technology for green hydrogen production (*3*–*5*).

However, the widescale adoption of PEMWE is hindered by economic challenges surrounding the high costs associated with electrolyzer production and operation. One area in critical need of improvement is the anode, where the oxygen evolution reaction (OER) occurs (*6*). The OER is an intrinsically sluggish reaction, with catalysts typically requiring large overpotentials to drive sufficient current densities, thereby requiring costly amounts of electricity input for operation. Additionally, the anode relies on expensive Ir-based electrocatalysts to drive sufficiently high and stable OER current densities in a harsh acidic and oxidizing environment.

Because of the expensive and scarce nature of Ir, a wealth of research has focused on improving the intrinsic activity and stability of Ir active sites within catalyst materials, with the goal of minimizing required loadings of Ir-based materials while also reducing electrical energy input needed to drive OER. Notable research effort focuses on developing and characterizing materials with reduced Ir composition, often leveraging a material system predominantly composed of cheaper, more abundant metals, with a small amount of Ir strategically incorporated (*7*–*15*). Another common strategy to improve the performance of Ir-based OER catalysts is to develop high-surface-area materials, or nanostructured materials, to maximize the total number of exposed Ir sites (*16*–*18*). This strategy often works to improve current magnitudes in non–diffusion-limited regimes, but does not necessarily improve the intrinsic activity of Ir active sites (*19*).

To understand if the intrinsic activity of Ir sites varies between catalyst materials, quantification of electrochemically accessible Ir sites is required. While this task may be feasible for pristine, flat thin films, there is a critical lack of robust methods to measure the quantity of electrochemically accessible Ir sites on metal oxide materials that are ubiquitous for oxidation reactions. H_2_ underpotential deposition (UPD) or CO oxidation voltammograms can be used to estimate quantity of active sites on certain model metallic surfaces such as Pt (*20*–*22*). However, these techniques are not applicable with many surfaces that do not adsorb H or CO, including most oxides, which are present under OER conditions (*23*). Alternate measures of geometric electrode area, Brunauer-Emmett-Teller (BET) surface area, mass loading, and electrochemical double-layer capacitance are instead used as normalization factors for standardizing OER current.

However, each of these factors is critically different from a true active site count and fails to effectively normalize current to yield an accurate estimate of turnover frequency (TOF) and intrinsic activity of a catalyst. BET surface area naturally overestimates the electrochemically accessible surface area (ECSA), representing total physical surface area of a powder, giving a theoretical maximum possible surface area of a catalyst on an electrode. The BET surface area normalization factor also fails to account for commonly occurring surface area reconstructions or electrode degradation processes under reaction conditions over time (*14*, *24*, *25*). Mass loading corrects for total quantity of catalyst or total Ir present on the electrode, but fails to account for surface area or to distinguish surface from core Ir sites. Furthermore, OER current normalized by mass loading is commonly a function of the degree of loading itself, with mass-normalized OER current artificially reduced for electrodes with high mass loadings, due to the diffusion limitations with high loadings (*26*, *27*).

Electrochemical double-layer capacitance is often estimated as a proportional measure of ECSA and is commonly used as a normalization constant for OER current (*13*, *28*–*31*). While electrochemical double-layer capacitance offers a more direct measurement of ECSA than BET surface area or mass loading, it has critical limitations as a metric for active site count. This technique provides a measure of the double-layer capacitance for the entire electrode, which can consist of multiple material components, and not just the active area for OER. Additionally, conversion between double-layer capacitance and ECSA requires the use of a specific capacitance value, reported values of which are limited, and those estimates that are reported can span two or three orders or magnitude for a single material (*32*, *33*), creating notable errors and limiting the comparability of results within literature. As research continues to focus on materials with reduced Ir content, capacitance measurements fail to account solely for electrochemical surface Ir (ECSI) accessibility on a material surface and therefore lose relevance for tracking Ir sites with mixed-metal systems, where ECSA deviates from ECSI. Last, capacitive current can be convoluted with current from other competing processes, including faradaic processes, ion adsorption, and corrosion (*17*, *34*–*37*).

Alternatively, mercury UPD has been recently proposed as a method to directly measure the quantity of electrochemically accessible Ir sites on an electrode (*38*). Mercury UPD (and stripping) involves the selective deposition of a monolayer of Hg^0^ onto Ir sites from a dilute concentration of Hg^2+^ ion in electrolyte. The understanding that Hg selectively electrochemically adsorbs onto Pt-group metal sites (in their metal or oxide forms) has long been established (*39*–*44*), but only recently have researchers started leveraging the phenomenon for ECSI quantification on electrocatalysts (*38*, *45*–*47*).

However, there are critical complexities with the mercury UPD phenomenon that require deconvolution to accurately extract an underlying quantity of Hg deposited and stripped from a surface (*48*, *49*). The Hg UPD and stripping process involves several redox reactions, in addition to a chemical reproportionation step. There is therefore a notable discrepancy between the current magnitude directly observed in a cyclic voltammogram (CV) associated with Hg reduction and oxidation, and the actual quantity of Hg deposited/stripped as observed with an electrochemical quartz crystal microbalance (EQCM) (*48*). Existing protocols for ECSI quantification with Hg UPD have not accounted for the full complexity of Hg UPD processes or rectified this discrepancy between electrochemical current and physical EQCM results. A lack of characterization of Hg interactions with noncatalyst components of electrodes, such as carbon-based supports and substrates, adds further complexity for researchers analyzing Hg UPD CVs and can lead to erroneous interpretations. Existing protocols are also critically limited by inconsistency and inaccuracy of redox peak identification and selection for Hg-Ir monolayer deposition and stripping, an all-important capability for Hg UPD Ir site quantification. Given these limitations, there is a critical need for careful characterization of Hg processes that accurately accounts for the full complexity of the system. By fulfilling this need, we herein enable the development of an Ir quantification technique that is based in the proof of its underlying physical phenomena and chemistry.

Here, we take an enhanced approach to existing Hg UPD protocols and deconvolute the complex mercury reaction network using a rotating ring disk electrode (RRDE) to characterize the net quantity of Hg deposited and stripped throughout CVs onto and off the Ir surface sites. We show excellent reversibility for the deposition and stripping of Hg, corroborating previously reported EQCM results (*48*). We identify critical variables that affect the nature of the deposition and stripping process, such as potential ranges and rotation rates. We propose a procedure and analysis for researchers to be able to quantify the ECSI of electrodes that is convenient, quick, and robust and can be performed in between other electrochemical tests. We see great potential and merit to using the technique for characterizing changes in electrochemical accessibility of Ir, particularly for the many dynamic materials studied that undergo surface structure and composition changes throughout testing (*7*, *10*, *13*–*15*, *50*–*52*). We demonstrate that our Hg UPD and stripping procedure and analysis effectively normalizes OER current for benchmarked IrO*_x_* to a single universal curve based on accessible Ir sites for a wide range of mass loadings.

## RESULTS

### Mercury deposition on carbon and Ir-based surfaces

Mercury UPD and stripping as an ECSI quantification technique involves selectively quantifying the amount of Hg that electrochemically deposits onto and strips off the surface Ir sites of a material in a monolayer or semi-monolayer fashion throughout a CV. Commonly used rotating disk electrodes for the OER in an aqueous media system typically consist of a conductive glassy carbon (GC) substrate with catalyst particles bound to the surface, a polymeric conductive binder (most commonly Nafion), and often a high-surface-area conductive support (such as Vulcan carbon). Throughout this work, we investigate mercury deposition and stripping using a characteristic electrode composed of a GC disk substrate with particulate amorphous IrO*_x_* as a benchmarked iridium oxide catalyst material and a Vulcan carbon support. Because these electrodes are composed of multiple components, it is critical to first identify and characterize the features and regions of a CV that correspond to interactions between mercury and the various electrode materials, such as GC and Vulcan carbon support.

To conduct electrochemical mercury deposition and stripping on materials, a non-interacting supporting electrolyte, such as 0.1 M HClO_4_, is used. CVs are performed by cathodically sweeping from an initial voltage of 1.2 V_NHE_ (voltage versus the normal hydrogen electrode) to a selected minimum vertex potential point, and then reversing the scan anodically back to 1.2 V_NHE_. After collecting a background CV in the 0.1 M HClO_4_ electrolyte, a small amount of Hg(NO_3_)_2_ is added to the electrolyte as the Hg^2+^ source to create a final solution with 1 mM Hg(NO_3_)_2_. The difference between these two CVs therefore shows the features associated with mercury redox events and deposition/stripping of mercury onto/off the electrode material surfaces.

**Fig. 1.**
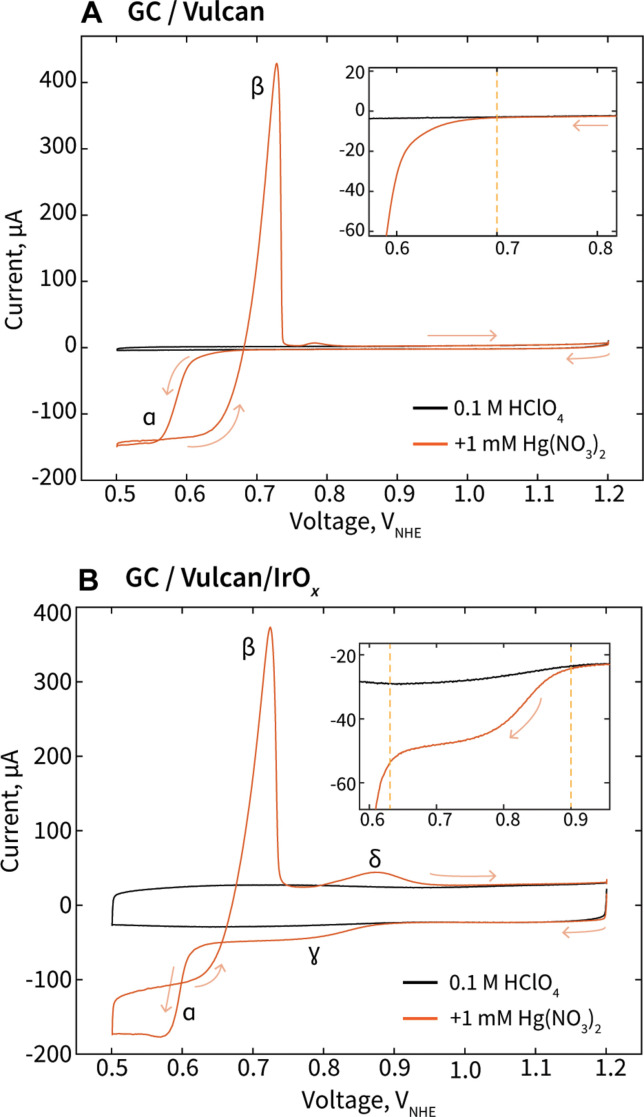
CVs of electrodes in acidic electrolyte showing key mercury interactions and electrochemical processes. (**A**) CV of GC disk electrode with Vulcan carbon (GC/Vulcan) in 0.1 M HClO_4_, with and without 1 mM Hg(NO_3_)_2_. Arrows indicate voltage sweep direction, with the CV beginning at 1.2 V_NHE_. Inset: Magnified view of the initial cathodic sweep, where the CVs begin to deviate as mercury reduction commences, with a dotted line at 0.7 V_NHE_ indicating the point of deviation. (**B**) CV of GC disk electrode with Vulcan carbon and IrO*_x_* (40 μg_Ir_/cm^2^) in 0.1 M HClO_4_, with and without 1 mM Hg(NO_3_)_2_. Inset: Magnified view of the initial cathodic sweep in the Hg UPD region, ɣ, where Hg deposition on Ir starts. Dotted lines indicate where the UPD region, ɣ, begins and ends.

When assessing the CVs of GC with only Vulcan carbon support (“GC/Vulcan”), we observe that the initial cathodic sweep in the system with added Hg starts to deviate at 0.7 V_NHE_ (Fig. 1A, inset), where Hg deposition or condensation begins on the electrode’s various carbon surfaces, after which a limiting reducing current is reached at feature ɑ, ~0.62 V_NHE_ (Fig. 1A). On the anodic return, a large anodic stripping peak β occurs at ~0.71 V_NHE_, associated with the electrochemical removal of Hg from the electrode’s carbon surfaces (*49*, *53*).

When conducting the same protocol for an electrode composed of a GC disk, Vulcan carbon support, and IrO*_x_* (40 μg_Ir_/cm^2^) (“GC/Vulcan/IrO*_x_*”), the resulting CV is similar, yet with critical differences (Fig. 1B). For the GC/Vulcan/IrO*_x_* electrode, there is now an earlier deviation, labeled as feature ɣ, in the cathodic sweep starting at ~0.9 V_NHE_, where Hg begins to electrochemically deposit onto Ir-based surfaces in the “underpotential region” before bulk, multilayer Hg deposition and condensation begins at 0.62 V_NHE_ (Fig. 1B, inset). On the anodic sweep, we observe peak β, the Hg stripping peak consistent with the GC/Vulcan system observed. We note that peak β, in addition to representing bulk Hg stripping from carbon surfaces (*54*), may also capture some bulk stripping from Hg multilayers on Ir, as this peak was similarly observed previously when performing Hg deposition and stripping with a pure Ir metal disk RDE in identical electrolyte (*49*). The additional anodic peak δ, at ~0.88 V_NHE_, is the stripping of Hg from Ir surfaces, as this peak only appears when IrO*_x_* is present on the electrode. This stripping peak δ, in addition to feature ɣ in the Ir-Hg UPD region, is of key interest as we work to establish an ECSI quantification technique with Hg deposition and stripping.

UPD is technically defined as small amounts of atoms on a foreign surface at potentials more positive than those predicted by the Nernst equation (*55*). As we will explore throughout this work, the “UPD” region associated with peak ɣ actually begins at more cathodic potentials of the minimum thermodynamic potential of 0.939 V_NHE_ for mercury deposition. Most of peak ɣ lies within the thermodynamically predicted potential range, making the term UPD a bit of a misnomer. We note that while the standard redox for mercury deposition is 0.850 V_NHE_, once accounting for the low 1 mM concentration of Hg^2+^ within the solution, the calculated redox of mercury deposition within our solution is 0.939 V_NHE_. Nevertheless, as we will show throughout this work, the “underpotential” deposition peak ɣ (after deconvolution of Hg reactions) displays typical behavior associated with UPD, as it represents monolayer or sub-monolayer deposition Hg from Ir surfaces, before bulk Hg^0^ deposition and condensation begins at ɑ.

### Competing reaction routes for mercury reduction and oxidation

The nature of electrochemical mercury deposition and stripping is a complex process that is highly dependent on electrolyte environment, electrode composition, and fluid dynamics of an electrochemical system. Mercury deposition and stripping in aqueous, acidic, non-interacting electrolytes has been studied on several materials (*56*, *57*), with the following redox and chemical reactions identified:

Standard reduction/oxidation reactions

$$2\;{\text{Hg}}^{2+}+\text{2e}-⇌{{\text{Hg}}_{2}}^{2+}\;\left({\text{Hg}}^{2+}+\text{1e}-⇌{\text{Hg}}^{1+}\right)\;E^{0}=0.911\;V_{\text{NHE}}$$ (1)

$${\text{Hg}}^{2+}+\text{2e}-⇌{\text{Hg}}^{0}\;E^{0}=0.850\;V_{\text{NHE}}$$ (2)

$${{\text{Hg}}_{2}}^{2+}+\text{2e}-⇌2\;{\text{Hg}}^{0}\;({\text{Hg}}^{1+}+\text{1e}-⇌{\text{Hg}}^{0})\;E^{0}=0.796\;V_{\text{NHE}}$$ (3)

Reproportionation reaction

$$\begin{array}{c} {\text{Hg}}^{0}+{\text{Hg}}^{2+}⇌{{\text{Hg}}_{2}}^{2+}\;({\text{Hg}}^{0}+{\text{Hg}}^{2+}⇌2\;{\text{Hg}}^{1+})\\ K_{\text{eq}}=167\;\left(\text{highly irreversible}\right) \end{array}$$ (4)

**Fig. 2.**
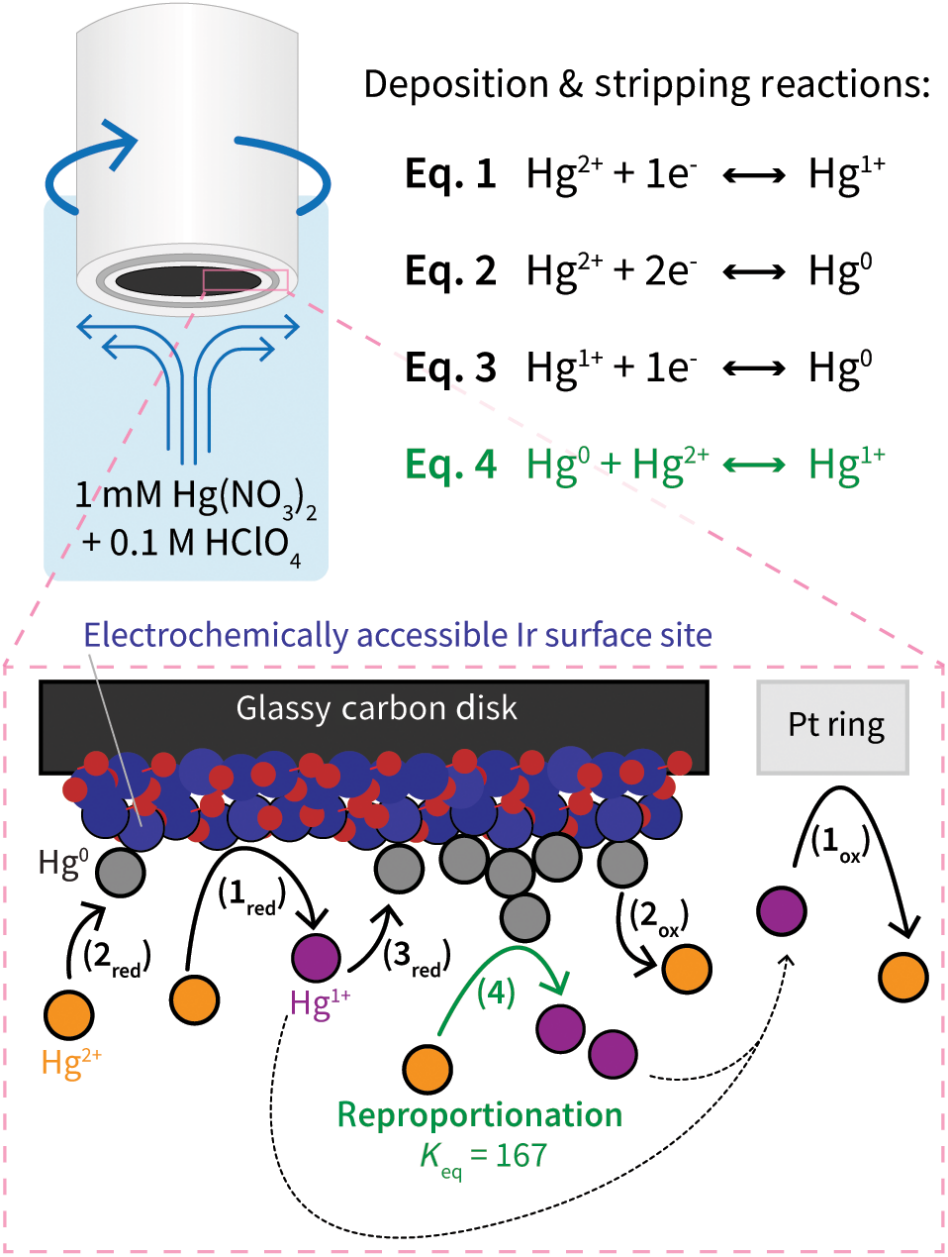
Schematic of the RRDE used throughout this work, and the possible Hg deposition and stripping reactions throughout a Hg UPD CV. Electrochemical reactions are shown in black, while the chemical reproportionation step is shown in green. Subscripts for each step indicate the direction of the electrochemical reaction (red, reduction; ox, oxidation).

For clarity and stoichiometric convenience, we will refer to each atom in a Hg_2_^2+^ dimer as a Hg^1+^ species throughout this work, with simplified versions of reactions in terms of Hg^1+^ in parentheses shown above. Overall, there are three possible competing electrochemical redox reactions that can occur when performing Hg UPD CVs, in addition to a highly irreversible and “electrochemically silent” chemical reproportionation Eq. 4, whereby reduced Hg^0^ can interact with Hg^2+^ to form Hg^1+^. Given the large *K*_eq_ value for the reproportionation step, Hg^1+^ (i.e., a Hg_2_^2+^ dimer) is very stable against disproportionation in solution (*57*). While we use an electrolyte solution that only supplies Hg^2+^ ions initially, throughout the CV it is possible to create Hg^1+^ through a few separate reaction routes. First, Hg^1+^ can be created by directly reducing Hg^2+^ electrochemically through Eq. 1. Alternatively, Hg^2+^ can be electrochemically reduced to Hg^0^ through Eq. 2, which can then undergo reproportionation through Eq. 4 to yield Hg^1+^. Acting as both an intermediate and product, Hg^1+^ can also be formed through Eq. 1, can be reduced to Hg^0^ through Eq. 3, and can undergo reproportionation back to Hg^1+^ through Eq. 4. A schematic of these reactions for Hg deposition onto and stripping off the Ir sites in an RRDE system is provided in Fig. 2.

To track and deconvolute the possible mechanisms at play throughout our mercury deposition studies, we use a highly sensitive RRDE equipped with a Pt ring that can probe the local concentration of Hg^1+^ formed at the disk throughout testing. The Pt ring is held at the oxidizing potential of 1.3 V_NHE_ to oxidize any Hg^1+^ ions to Hg^2+^ through Eq. 1, and therefore acts as a probe for Hg^1+^ production at the disk.

**Fig. 3.**
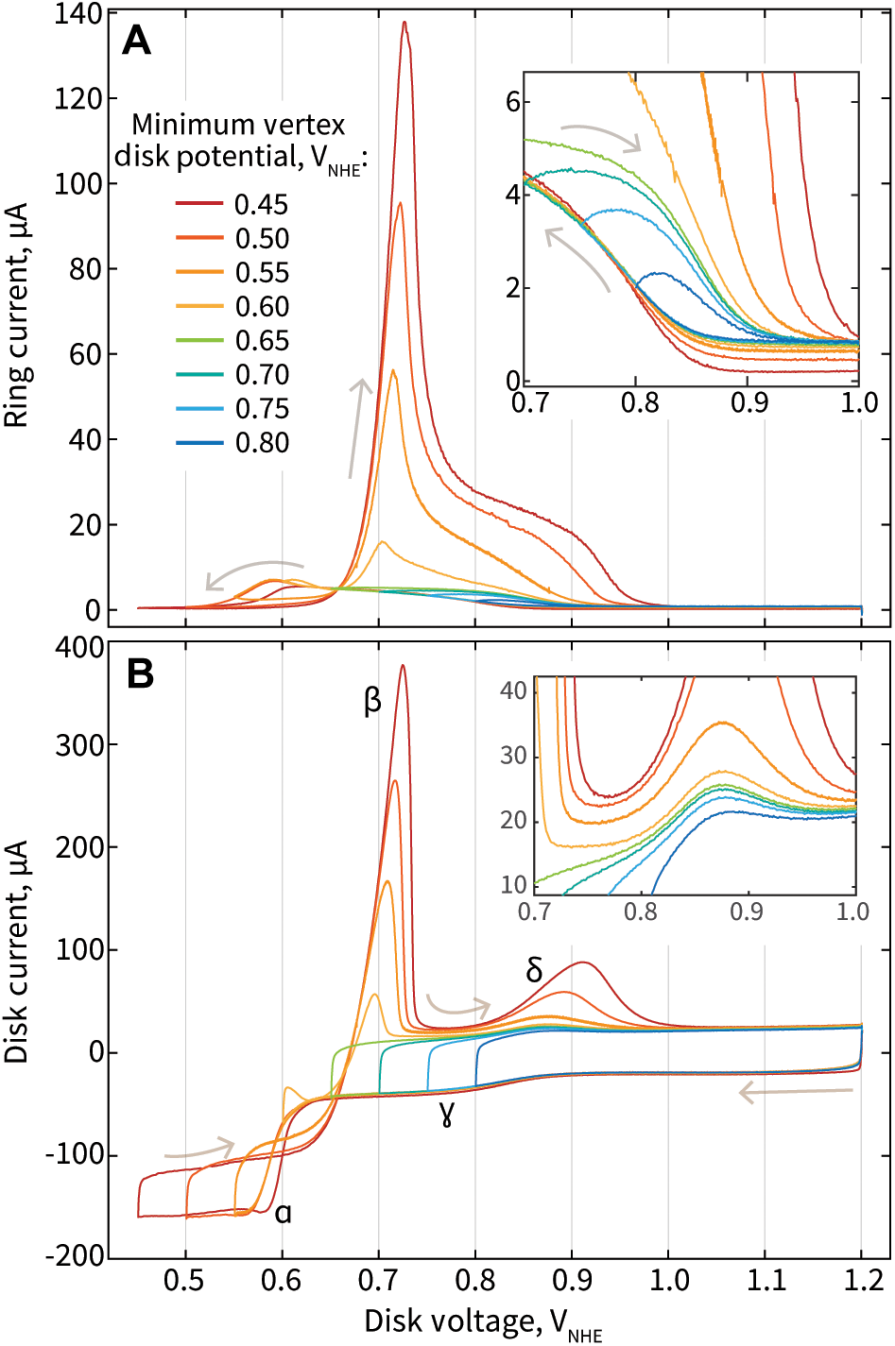
CVs of a GC/Vulcan/IrO*_x_* electrode in Hg-containing electrolyte with varying minimum vertex disk potential conditions. (**A**) Ring current and (**B**) disk current versus disk voltage of an RRDE with a Pt ring and GC disk electrode with Vulcan carbon and IrO*_x_* (40 μg_Ir_/cm^2^) in 0.1 M HClO_4_ with 1 mM Hg(NO_3_)_2_. (A, inset) Magnified view of ring currents from 0.7 to 1.0 V_NHE_. (B, inset) Magnified view of peak δ.

Hg deposition and stripping mechanisms on a GC/Vulcan/IrO*_x_* electrode were investigated by using an RRDE and conducting CVs with increasingly cathodic minimum potentials. As the minimum vertex potential is decreased in sequential CVs from 0.8 to 0.45 V_NHE_, we observe the appearance and growth of key features in the CV at the disk, as well as in the ring response (Fig. 3). For all CVs, as we sweep cathodically, we observe that the formation of Hg^1+^ (as indicated by peaks in the ring current) starts at ~0.86 V_NHE_ (Fig. 3A, inset), just after the ɣ feature in the disk current indicating Hg UPD on Ir, which begins at 0.9 V_NHE_. When the minimum vertex potential of the CVs is kept above 0.6 V_NHE_, the magnitude of the Ir-Hg stripping peak δ at the disk is very small and maintains similar magnitude despite variations in the low potential limit of the CV (Fig. 3B, inset). This observation is consistent with the understanding that Hg deposits in a monolayer fashion at potentials anodic of 0.6 V_NHE_, and therefore, its stripping peak maintains a consistent magnitude. The corresponding ring current for these CVs increases steadily as the disk potential is swept cathodically and then decreases as the disk potential is swept back anodically over the underpotential regime (Fig. 3A, inset). The ring current does not show any obvious peak mirroring the Ir-Hg stripping peak at the disk, and therefore, we understand that, as Hg strips from Ir, it follows Eq. 2.

Alternatively, when the low potential limits of the CV are more cathodic than 0.6 V_NHE_, there is a clear growth of the Ir-Hg stripping peak δ with increasingly cathodic minimum vertex potentials. Because potentials below 0.6 V_NHE_ cause Hg to be deposited in bulk (indicated by peak ɑ), the minimum vertex potential and longer time exposed to cathodic potentials results in the deposition of increasingly thick bulk layers of Hg at Ir-based surfaces, resulting in the growth of the Ir-Hg stripping peak δ as a function of the low potential limit. During the cathodic sweep of these CVs, the ring current increases before reaching a maximum at ~0.6 V_NHE_, after which it decreases to zero. On the anodic return, a large and sharp peak in the ring current appears at ~0.71 V_NHE_, mirroring peak β at the disk associated with bulk Hg stripping from carbon surfaces. This mirroring indicates that the bulk stripping of Hg at the disk (peak β) largely proceeds through Eq. 3, or bulk Hg^0^ may physically dislodge from the carbon surfaces, rapidly undergoing reproportionation to Hg^1+^ once in electrolyte. As the lower vertex potential of the CV is extended cathodically, we also observe the growth of a second peak at ~0.87 to 0.9 V_NHE_ in the ring current, mirroring the growing Ir-Hg stripping peak δ at the disk. This observation indicates that as an increasingly bulk Hg layer is stripped from Ir surfaces at δ, the stripping of these bulk layers may proceed through Eq. 3, or through bulk Hg^0^ dislodgement from the Ir surface, followed by rapid reproportionation Eq. 4 to Hg^1+^ in electrolyte. This is in contrast to monolayer Ir-Hg stripping that proceeds through Eq. 2.

### Bulk-type versus monolayer-type Hg coverage

**Fig. 4.**
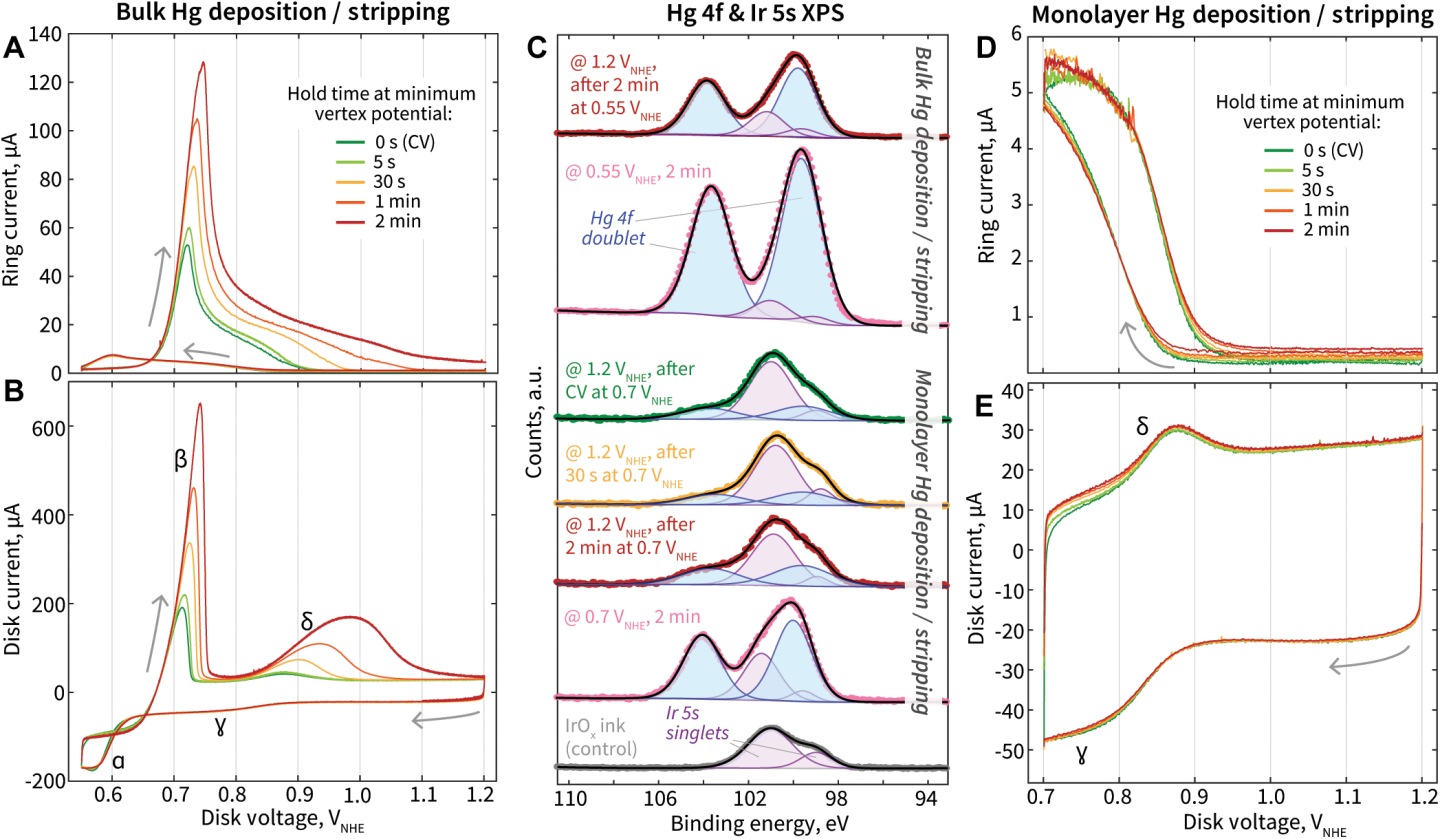
Electrochemical and surface composition characterization of GC/Vulcan/IrO*_x_* electrodes exposed to various minimum vertex potential and time conditions. (**A**) Ring current and (**B**) disk current versus disk voltage with varying extended time at the lower potential condition of 0.55 V_NHE_, where bulk deposition occurs. The CVs are conducted with a disk electrode consisting of a GC disk with Vulcan carbon and IrO*_x_* (40 μg_Ir_/cm^2^) in 0.1 M HClO_4_ with 1 mM Hg(NO_3_)_2_. (**C**) Hg 4f and Ir 5s (deconvoluted) XPS spectra for IrO*_x_* (40 μg_Ir_/cm^2^) (without Vulcan carbon) electrodes exposed to varying potentials for varying amounts of time. The applied potential under which the electrode is taken out of the electrolyte is indicated with the @ symbol. Samples exposed to bulk versus monolayer Hg deposition/stripping conditions are labeled respectively. An IrO*_x_* control spectrum is included for the identification of the Ir 5s peaks. (**D**) Ring current and (**E**) disk current versus disk voltage with varying extended time at the lower potential condition of 0.70 V_NHE_, where monolayer deposition occurs.

Assessing the disk CVs with varying minimum vertex potentials and their associated ring currents, we can categorize the CVs as those involving bulk-type versus monolayer-type Hg deposition/stripping. To further probe these deposition/stripping behaviors as a function of applied deposition time, we selected two minimum vertex potentials, 0.7 and 0.55 V_NHE_, and performed CVs with extended holds from 0 s to 2 min at the minimum vertex potential, before resuming the anodic sweep to 1.2 V_NHE_ (Fig. 4, A and B). When CVs in the Hg bulk deposition/stripping regime experience increasing hold times at 0.55 V_NHE_, there is clear growth and distortion of the Hg stripping peaks off both carbon (β) and Ir (δ). Essentially, as more time is spent at 0.55 V_NHE_ depositing Hg in bulk on carbon and Ir surfaces, greater magnitude stripping peaks are observed as a result. However, for the Hg monolayer deposition/stripping regime with a minimum vertex potential of 0.7 V_NHE_, the Ir-Hg stripping peak does not appreciably change in magnitude as hold time at 0.7 V_NHE_ is increased, consistent with behavior expected for monolayer-type coverage of an adsorbate (Fig. 4, D and E). Similarly, the quantity of Hg^1+^ species detected during the CV, as measured by the ring current, is not affected by the length of hold at the low potential limit.

X-ray photoelectron spectroscopy (XPS) is used to qualitatively assess the extent of Hg deposition and efficacy of stripping from the electrode in both cases—the Hg “bulk”-type and “monolayer”-type CVs (Fig. 4C). Samples are prepared by exposing electrodes to specific electrochemical conditions and washing with Milli-Q water after removal from the electrolyte. The Hg 4f and Ir 5s XPS spectra overlap in binding energy; to clarify contributions from these elements, IrO*_x_* species are fitted with two distinct singlets (purple) and Hg species are fitted with one doublet (blue) consistent with Hg^0^ (*58*). To first assess the samples associated with the bulk Hg CV with a minimum vertex potential of 0.55 V_NHE_, one sample is prepared by removing the working electrode under potential control at 0.55 V_NHE_ after the cathodic CV sweep and a 2-min hold at this minimum vertex potential. Alternatively, another sample is prepared by removing the working electrode under potential control at 1.2 V_NHE_, after completing the full CV with a 2-min hold at 0.55 V_NHE_. The XPS spectrum of the sample removed at 0.55 V_NHE_ shows a notable quantity of Hg present on the electrode surface, consistent with the understanding that bulk Hg is deposited at 0.55 V_NHE_ throughout the hold time. In contrast, the XPS spectrum of the sample removed at 1.2 V_NHE_ after the complete CV shows a reduced quantity of Hg on the electrode surface, as expected after experiencing stripping of the bulk Hg. However, a notable amount of Hg remains present on the surface even after undergoing electrochemical stripping. When Hg is deposited in bulk, it may be difficult for the electrode to completely strip the Hg from its surface, either from carbon or Ir sites.

This sample preparation was repeated for monolayer Hg CVs with a minimum vertex potential of 0.7 V_NHE_, with additional samples removed at 1.2 V_NHE_ after CVs with potential holds at 0.7 V_NHE_ for 0 s (normal CV) and 30 s. As observed in the XPS spectrum of the sample removed under potential control at 0.7 V_NHE_ after a 2-min hold, there is notably smaller quantity of Hg present on the surface compared to the sample removed under 0.55 V_NHE_ (Fig. 4C). This observation is consistent with the understanding that the 0.55 V_NHE_ sample experiences bulk Hg deposition, while the 0.7 V_NHE_ sample experiences monolayer-type deposition. XPS spectra of the samples held at 0.70 V_NHE_ for varying amounts of time, followed by the anodic return to 1.2 V_NHE_, show notably less Hg present compared to all other samples. Despite these samples spending increasing length of time at 0.7 V_NHE_, Hg is stripped with a similarly high efficacy from the electrode surfaces on these samples, with minimal Hg still present in their spectra after stripping.

We have shown mercury to deposit in a monolayer-type nature and to be largely reversible, with high efficacy of stripping when CVs with a minimum vertex potential of 0.7 V_NHE_ are used. The minimum vertex potential of 0.7 V_NHE_ was also selected because this is the lowest potential possible before Hg condensation on carbon surfaces begins (Fig. 1A, inset). For the remainder of this work, we delve further to understand how to leverage these CVs with a minimum vertex potential of 0.7 V_NHE_ for quantifying monolayer-type mercury deposition and stripping.

### Deconvolution of Hg deposition and stripping current in the monolayer regime

As shown in Figs. 3 and 4 (A and B), the portion of the Hg UPD CV that is cathodic of 0.9 V_NHE_ is accompanied with a notable amount of ring current, indicating the production of Hg^1+^ species. Given the presence of Hg^1+^, Hg deposition and stripping through Eq. 2 must be occurring simultaneously to other Hg reactions that produce Hg^1+^ (i.e., Eqs. 1 and 4 in the forward direction, and Eq. 3 in the reverse direction). Additionally, it is possible that Hg deposition not only proceeds through a single step as Eq. 2 but may also involve two individual redox steps of Eq. 1 and then Eq. 3, creating Hg^1+^ as an intermediary ion in electrolyte, which may be further reduced and deposited onto an Ir site as Hg^0^, or may be transported by the electrolyte flow field and detected at the ring.

Furthermore, the chemical reproportionation step (Eq. 4) may occur as Hg^0^ is deposited onto Ir sites in a monolayer fashion such that incoming Hg^2+^ ions from electrolyte interact with the Hg^0^ to undergo reproportionation and form Hg^1+^ ions. This reproportionation step (Eq. 4), which strips deposited Hg^0^ through a chemical step, can be expected to occur in an equilibrium with the Hg^0^ deposition processes simultaneously occurring, since the Ir-Hg electrochemical stripping peak magnitude is insensitive to additional time held at 0.7 V_NHE_. In summary, simultaneous electrochemical Hg deposition and chemical stripping through reproportionation is expected to occur in a stable equilibrium to maintain a consistent amount of Hg being electrochemically stripped from Ir sites on the anodic return of the CV despite elongated exposure to a minimum vertex potential condition of 0.7 V_NHE_. As noted before, the stripping peak in the disk CV is not mirrored in the ring current, and therefore, the electrochemical stripping of Hg must occur through Eq. 2.

To find the current resulting only from net electrochemical Hg deposition and stripping, we must separate current forming Hg^1+^ ions from that responsible for Hg^2+^ ⇌ Hg^0^ processes. Regardless of the specific reaction route taken, the total number of electrons transferred at the disk to produce Hg^1+^ ions is equal to the total number of electrons transferred at the ring to oxidize them back to Hg^2+^ (after accounting for the ring collection efficiency, *N*). Therefore, to calculate the quantity of current representing the equilibrium amount of Hg deposited (negative current) or stripped (positive current) from Ir sites at each voltage step, *I*_Hg_, we can use Eq. 5

$$I_{\text{Hg}}=I_{\text{disk},\text{Hg}}+\left(\frac{I_{\text{ring},\text{Hg}}-I_{\text{ring},\text{Hgbkg}}}{N}\right)-I_{\text{disk},0.1\text{MHClO}4}$$ (5)

where *I*_disk,Hg_ is the disk current with added Hg in electrolyte, *I*_ring,Hg_ is ring current with added Hg in electrolyte, *I*_ring,Hgbkg_ is the background ring current in Hg-added electrolyte when no Hg^1+^ is being produced at the disk, *N* is the collection efficiency of the ring for Hg^1+^, and *I*_disk,0.1MHClO4_ is the disk current in 0.1 M HClO_4_ without Hg. The cumulative integration of *I*_Hg_ along time throughout a CV therefore represents the charge passed for net Hg deposition and stripping. Net electrochemical deposition and stripping of a Hg monolayer on Ir sites occurs when *I*_Hg_ is negative and positive, respectively. Deposition occurs throughout the cathodic sweep of the CV starting around ~0.9 V_NHE_ until the minimum vertex potential and continues during the anodic return sweep until ~0.75 V_NHE_ (where *I*_Hg_ crosses zero and where the minimum of the associated cumulative charge curve). Stripping occurs on the anodic sweep at potentials greater than ~0.75 V_NHE_. On the basis of experimental Hg UPD CV curves and positions of the Hg deposition and stripping features, we determine integration bounds of the cumulative charge curve to be from 0.95 V_NHE_ on the cathodic sweep to 1.05 V_NHE_ on the anodic sweep (fig. S1). The difference between the cumulative charge at 0.95 V_NHE_ on the cathodic sweep and the minimum of the cumulative curve represents the quantity of Hg deposited, while the difference between the cumulative charge curve at 1.05 V_NHE_ on the anodic sweep and the minimum of the cumulative charge curve represents the quantity of Hg stripped. Considering the XPS results, as well as previously reported EQCM characterization of Ir electrodes (*48*), this deposition/stripping process is expected to be highly reversible. However, performing this calculation using an empirically determined *N* (0.382) from a ferrocyanide/ferricyanide redox results in a lack of reversibility, with the calculated charge from Hg deposition being notably greater than that from Hg stripping (fig. S2). Given that this analysis disagrees with ours and others’ established understanding that the Hg deposition and stripping process is highly reversible (*48*), we explore this discrepancy and provide a relevant, more accurate analysis of the Hg^1+^ collection efficiency specific to this system, by revisiting how *N* is determined.

### Hg^1+^ ring collection efficiency determination

*N* for RRDE systems is commonly empirically measured with a ferrocyanide/ferricyanide half reaction, determined as the ratio of anodic ring limiting current to the cathodic disk limiting current. When conducting this method to determine *N* of our RRDE, an empirical value of 0.382 was determined (table S1 and fig. S3). We note that this method of determining *N* is based on key assumptions for ideal systems and may not be transferable to all RRDE systems and electrolyte chemistries. For example, the ferrocyanide/ferricyanide *N* determination method assumes that the reaction at the ring “proceeds under pure diffusion control,” resulting from an appropriately and sufficiently oxidizing voltage at the ring to rapidly reverse the cathodic reaction at the disk (*59*). To determine an appropriate voltage for Hg^1+^ oxidization to Hg^2+^ in our system, we varied the applied ring potentials of 1.1 to 1.6 V_NHE_ and conducted multiple Hg UPD CVs. The ring potential has a notable effect on the ring current magnitude observed and is not able to reach a purely diffusion-controlled regime before competing OER current is observed (fig. S4). Therefore, we selected an applied ring potential of 1.3 V_NHE_ (1.365 V_RHE_) with which to conduct all of our Hg UPD experiments, yielding relatively high ring currents for Hg^1+^ oxidation, while minimizing OER current and expected Pt dissolution (*60*). However, this voltage is not sufficiently oxidative to yield the absolute maximum, diffusion-controlled current from the Hg^1+^ ⇌ Hg^2+^ redox, and therefore, the traditional *N* determination from the ferrocyanide/ferricyanide system cannot be directly applied.

Additionally, given the high-surface-area IrO*_x_* and Vulcan carbon support on the GC disk for our electrodes, this roughened surface morphology on the working electrode may cause slight turbulence or deviations from the ideal laminar flow at the RRDE surface, causing reductions in *N* that may vary sample to sample. To test the impact of disk surface roughness for this system, we performed measurements of *N* using the ferrocyanide/ferricyanide method, with IrO*_x_* and Vulcan carbon support drop-casted onto the GC disk. The determined *N* is slightly smaller when using this sample, in the range of 0.32 to 0.37, depending on rotation rate and limiting currents selected (table S2 and fig. S5). Again, these observations support the conclusion that a traditional empirical *N* determination from a ferrocyanide/ferricyanide system cannot be applied to this Hg system.

**Fig. 5.**
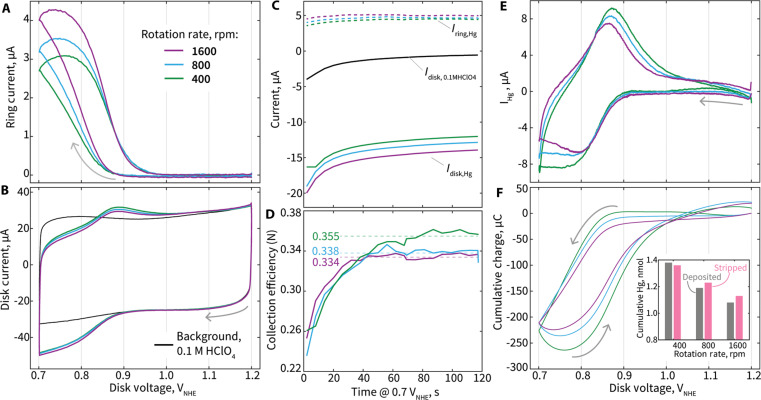
Electrochemical data and analysis for GC/Vulcan/IrO*_x_* electrodes following the recommended Hg UPD CV protocol under various rotation rates. (**A**) Ring current and (**B**) disk current versus disk voltage with varying rotation rate. The CVs are conducted with a disk electrode consisting of a GC disk with Vulcan carbon and IrO*_x_* (40 μg_Ir_/cm^2^) (GC/Vulcan/IrO*_x_*) in 0.1 M HClO_4_ with 1 mM Hg(NO_3_)_2_. The background CV is shown in black. (**C**) Ring and disk current for the GC/Vulcan/IrO*_x_* during an extended 2-min hold at 0.7 V_NHE_ in 0.1 M HClO_4_ with 1 mM Hg(NO_3_)_2_. The background disk current response for the electrode in 0.1 MHClO_4_ is shown in black. (**D**) Calculated collection efficiency (*N*) as a function of time during a 2-min hold on 0.7 V_NHE_ using Eq. 5. (**E**) Current (*I*_Hg_) associated with net electrochemical Hg deposition onto and stripping from Ir sites as a function of disk voltage. (**F**) Cumulative integrated charge from Hg deposition and stripping as a function of disk voltage. Inset: Cumulative mol of Hg deposited and stripped throughout the CVs conducted with various rotation rates. Legend of (A) applies to (A) to (F).

Alternatively, we use a sample-specific, direct measurement method to determine *N* for this Hg system. We leverage the knowledge that has already been established for our system during extended potential holds for time, *t*, at 0.7 V_NHE_, where the following relationship is expected

$$N\left(t\right)=\left|\frac{I_{\text{ring},\text{Hg}}-I_{\text{ring},\text{Hgbkg}}}{I_{\text{disk},\text{Hg}}-I_{\text{disk},0.1\text{MHClO}4}}\right|V=0.7\;V_{\text{NHE}}$$ (6)

where *I*_ring,Hg_ is the ring current in Hg-added electrolyte, *I*_ring,Hgbkg_ is the background ring current in Hg-added electrolyte when no Hg^1+^ is being produced at the disk, *I*_disk,Hg_ is the disk current in Hg-added electrolyte, and *I*_disk,0.1MHClO4_ is the disk current in baseline 0.1 M HClO_4_. A visual explanation of these variables for a characteristic Hg UPD CV is provided in fig. S6. To obtain these data, we conduct a cathodic sweep from 1.2 V_NHE_ to 0.7 V_NHE_ and hold the potential at 0.7 V_NHE_ for 2 min while recording *I*_ring,Hg_ and *I*_disk,Hg_. An identical experiment is performed for the same electrode in baseline 0.1 M HClO_4_ to determine *I*_disk,0.1MHClO4_. As the disk and ring currents quickly stabilize, *N*(*t*) is expected to reach a stable value, which is used as *N*. When performing this analysis for our GC/Vulcan/IrO*_x_* electrode, we calculate *N* at various rotation rates of 400, 800, and 1600 rpm, which yields *N* values between 0.334 and 0.355, slightly below the 0.382 number based on the ferrocyanide/ferricyanide redox (Fig. 5, C and D).

Equation 6 is based on our previously discussed findings, where simultaneous electrochemical Hg deposition and chemical stripping through reproportionation is expected to occur in a stable equilibrium at 0.7 V_NHE_. After complete filling of an equilibrated semi-monolayer, additional time at 0.7 V_NHE_ maintains a consistent quantity of Hg deposited on Ir, where the rate of electrochemically deposited Hg through Eq. 2 is equal to the chemically stripped Hg through Eq. 4. This understanding is corroborated by the consistent amount of electrochemical Hg stripping current observed on the anodic return of the CV regardless of the time duration for which the disk is held at 0.7 V_NHE_. Therefore, the disk current observed during an elongated hold at 0.7 V_NHE_ solely results from Hg^1+^ formation, either through direct formation through Eq. 1 at the disk or from Hg^0^ deposition through Eq. 2, followed by reproportionation through Eq. 4 to maintain net zero additional Hg deposition. For either reaction route, the number of electrons transferred at the disk to perform Hg reduction must equal the number transferred at the ring to perform Hg oxidation (after accounting for collection efficiency).

When incorporating this direct measurement for *N*, the calculated Hg deposition and stripping current throughout CVs demonstrate excellent reversibility. We calculate total quantities of Hg deposited and stripped during a CV consistently being within 10% of each other, corroborating previously reported EQCM-based findings (*48*).

### The effect of rotation rate on Hg deposition and stripping

The charge associated with deposition and stripping of Hg from Ir on these electrodes is also dependent on the RRDE rotation rate. As the RRDE rotation rate is increased, the magnitude of raw disk current throughout the most cathodic portions of the CV becomes greater, indicating a greater rate of Hg^2+^ reduction at the disk, as Hg^2+^ ions are more rapidly transported to the disk surface (Fig. 5B). Additionally, a greater magnitude of ring current is also observed with increasing RRDE rotation rates, indicating more rapid production of Hg^1+^ (Fig. 5A). Critically, after analyzing the raw currents and the amplitude of *I*_Hg_, net Hg deposition and stripping decreases as rotation rate is increased (Fig. 5E). This negative correlation between the net quantity of Hg deposited/stripped and the rotation rate can be explained by the role of the reproportionation step (Eq. 4) throughout the CVs. As rotation rate is increased, a greater flux of Hg^2+^ causes a greater reproportionation reaction frequency. Increased rotation rate can therefore be expected to yield a less complete monolayer on Ir surfaces, as Hg^2+^ reduced to Hg^0^ at the electrode is then more prone to destabilization through a reproportionation “attack” from a greater incoming flux of Hg^2+^ ions, causing both a decrease in net electrochemical Hg deposition and stripping. We note that, despite decreased net Hg deposition and stripping with higher rotation rates, excellent reversibility is still observed at any of the rotation rates examined.

The dependency of the monolayer occupancy on rotation rate is a critical observation, as it identifies a key variable to keep constant or account for when applying this technique to various electrodes. We estimate Hg monolayer occupancies on the IrO*_x_* surface of 0.32, 0.28, and 0.26 for rotation rates of 400, 800, and 1600, respectively. Monolayer occupancies are calculated based on the net quantity of Hg deposited and stripped throughout the Hg UPD CV, as well as Ir areal densities previously estimated using BET (table S3) (*61*, *62*). When determining which rotation rate is ideal to use for a Hg UPD protocol, we select and recommend 400 rpm, as this rotation rate yielded the greatest current amplitude and resolution of *I*_Hg_ current, had the greatest monolayer occupancy of Hg on Ir, and consistently demonstrated directly measured *N* values close to the theoretical maximum of 0.382 (Fig. 5D).

The optimized protocol we use and propose involves the Hg UPD CV at 400 rpm, followed immediately by a 2-min hold at 0.7 V_NHE_ to directly determine *N* of the electrode (Fig. 5C). This protocol is performed first in 0.1 M HClO_4_ electrolyte and repeated immediately after in 0.1 M HClO_4_ + 1 mM Hg(NO_3_)_2_. A detailed description of the recommended electrochemical protocol, a schematic of the protocol, and a description/identification of the critical variables needed from the electrochemical data for formal Hg UPD analysis are provided in the Supplementary Materials [“EC-lab (Potentiostat Control Software) Protocol”; fig. S6].

Alternative mathematical approaches to obtain measures of electrochemically accessible Ir site quantity were also explored, including using curves collected without rotation (0 rpm) and/or only accounting for Ir-Hg stripping peak magnitudes, as suggested in previous works (*38*, *45*). However, we did not find these methods to consistently achieve reliable or robust measures of electrochemically accessible Ir site quantity, providing further support for our 400 rpm protocol. More information on these approaches and their results is provided in the Supplementary Materials (“Alternative Ir-Hg Quantification and Mathematical Approaches”; figs. S7 and S8).

### Application of Hg UPD technique

We apply our Hg UPD ECSI quantification technique to electrodes with various loadings of IrO*_x_*—10, 20, 40, 80, and 160 μg_Ir_/cm^2^—to assess the efficacy of the Hg UPD site quantification technique for OER performance normalization (fig. S9). When accounting for electrode area, the total electrode mass loadings on each electrode can also be expressed as 2.4, 4.7, 9.5, 19, and 38 μg_Ir_. We prepare the electrodes with varied loadings by drop-casting the desired amount of IrO*_x_* ink onto the GC disk, with a constant amount of Vulcan carbon ink drop-casted on top. Hg UPD analysis is conducted at 400 rpm, followed by a measurement of electrochemical capacitance in the range of 1.0 to 1.2 V_NHE_ (fig. S10), and finally, a CV of 5 mV/s in the OER relevant potential regime is conducted at 1600 rpm to correlate this analysis with traditionally measured activity performance.

**Fig. 6.**
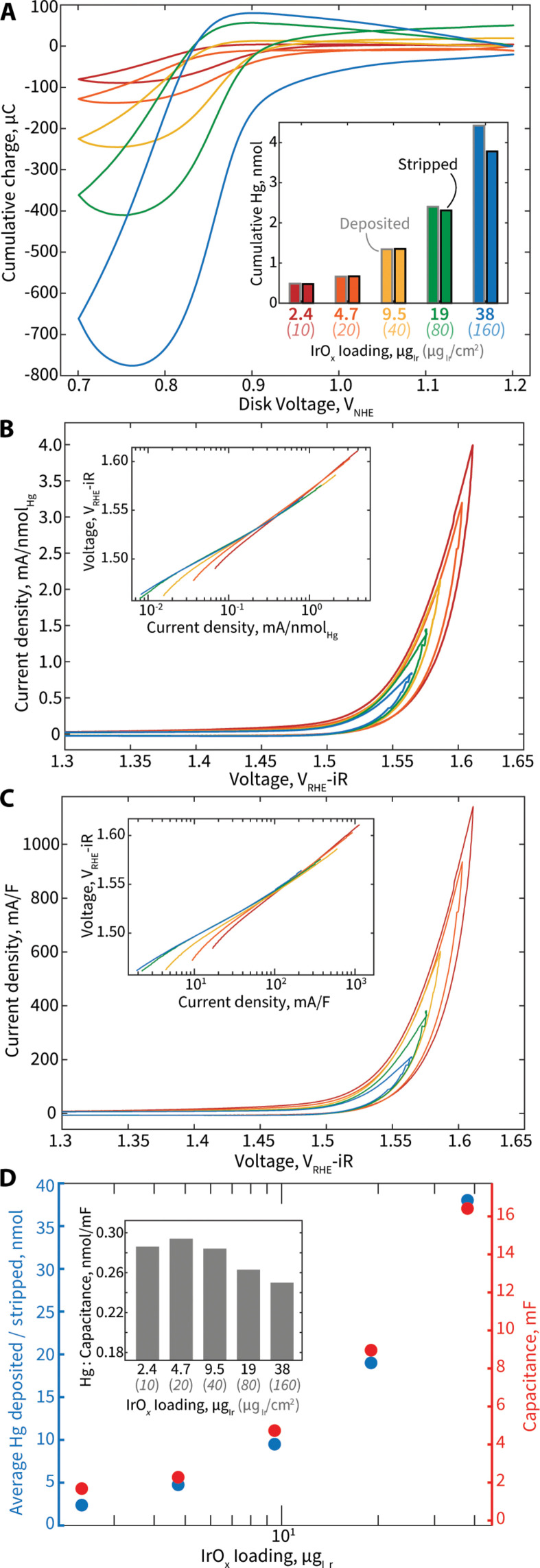
Hg UPD results, OER performance normalization, and comparison to conventional electrochemical capacitance results for GC/Vulcan/IrO*_x_* electrodes with varying amounts of Ir loading. (**A**) Cumulative charge associated with Hg deposition and stripping during a Hg UPD CV for electrodes with various loadings of IrO*_x_*, as specified in the inset. Inset: Cumulative quantity of Hg deposited and stripped during a Hg UPD CV for electrodes with various loadings of IrO*_x_*. The *x* axis labels of the inset serve as a legend for (A) to (C). (**B**) OER performance CVs, normalized by average amount of Hg deposited/stripped, for various loadings of IrO*_x_*. Inset: Associated Tafel plot for all samples. (**C**) OER performance CVs, normalized by electrode capacitance, for various loadings of IrO*_x_*. Inset: Associated Tafel plot for all samples. (**D**) Average quantity of Hg deposited and stripped (blue), as well as electrochemical capacitance (orange) versus electrode loading of IrO*_x_*. Inset: Ratio of quantity of Hg deposited/stripped to capacitance for electrodes with various IrO*_x_* loadings.

The Hg deposition and stripping charge versus disk voltage follows a consistent curve for all samples, with a clear increase in net amount of Hg deposited and stripped throughout the CVs mirroring the increase in IrO*_x_* loading (Fig. 6A). Excellent calculated reversibility of Hg deposition and stripping is observed for all samples, with the calculated reversibility of the Hg deposition and stripping marginally decreasing as IrO*_x_* loading is increased (Fig. 6A, inset). For the sample with the greatest loading of 160 μg_Ir_/cm^2^, the calculated irreversibility is 16%. A complete table of results and critical data for all electrodes is provided in the Supplementary Materials (table S4).

The mole quantity of Hg deposited and stripped throughout the Hg UPD CV is calculated by converting the average charge associated with Hg deposition and stripping, considering a two-electron deposition and stripping process. This quantity is used to normalize the OER performance CV current for each sample. OER activity CV curves normalized by mole Hg deposited/stripped show excellent normalization to a single universal curve, indicating that Hg deposition/stripping quantity acts as a suitable, robust measure of OER-relevant ECSI to reveal a consistent intrinsic Ir activity for these electrodes (Fig. 6B). This excellent normalization is also observed in the corresponding Tafel plot, which shows the normalized current averaged from the forward anodic and backward cathodic sweeps of the OER performance CV (Fig. 6B, inset).

The Hg monolayer occupancy factors are estimated as 0.45, 0.31, 0.31, 0.27, and 0.24 for the electrodes with 10, 20, 40, 80, and 160 μg_Ir_/cm^2^, respectively (table S5). As expected, monolayer completeness declines as IrO*_x_* loading increases and particle packing on the surface of the electrode becomes more crowded. As IrO*_x_* loading increases, an increasing fraction of the IrO*_x_* surface area is expected to become electrochemically inaccessible, resulting in lower calculated monolayer occupancy factors. On the basis of the electrode (10 μg_Ir_/cm^2^), which has the most optimal particle dispersion and minimized mass transfer limitations for the OER out of samples studied, we can roughly estimate a minimum Hg UPD conversion factor (or specific charge value) of ~85 μC_Hg_/cm^2^IrO*_x_*. We note that this factor value is likely an underestimate of the actual Hg UPD conversion factor, because the theoretical ECSA for this calculation is an overestimate as it is based on the total physical IrO*_x_* BET surface area applied to the electrode. This factor, although only an estimation, is similar to the previously observed conversion factor of 138.6 μC_Hg_/cm^2^_Ir_ for an Ir metal disk (*39*). When accounting for the differences in planar densities for Ir sites on IrO*_x_* and Ir metal, these estimates are highly consistent at 85 and 62 μC_Hg_/nmol_SurfaceIr_, respectively.

We additionally investigated the ability of the Hg UPD analysis to consistently normalize OER current of electrodes prepared with and without Vulcan carbon, using a loading of IrO*x* (40 μg_Ir_/cm^2^). When analyzing the Hg UPD CVs, we observe that the sample with Vulcan carbon yields greater Hg deposition and stripping current, with a greater Hg deposition and stripping cumulative charge (fig. S11). This result indicates that the addition of Vulcan carbon to the sample increases the ECSI quantity. Vulcan carbon acts as a high-surface-area, conductive network for the IrO*_x_* particles, improving their electrical connection to the GC substrate, thereby enabling improved electrochemical accessibility. While OER performance CVs show stark differences for these samples when normalizing by Ir mass loading, the CVs show consistent behavior when normalizing by Hg deposition and stripping cumulative charge (fig. S12), supporting the expectation that the intrinsic activity of the Ir sites is unchanged for these two electrodes. We note that the addition of Vulcan carbon to the electrode also improves the electrode’s ability to return to baseline current on the anodic sweep of the CV during Hg UPD.

We compare the Hg UPD OER normalization method to other commonly used methods, such as mass normalization and capacitance normalization. As expected, we observe that CVs normalized by Ir mass loading demonstrate higher OER current densities for samples with lowest loadings (fig. S13). This trend is identical when using BET surface area as a normalization factor, which is proportional to catalyst loading, and similarly assumes that the maximum possible amount of catalyst is exposed on the electrode to perform OER. The mathematical bias in OER performance when using mass or BET surface area as a normalization factor is widely observed and understood, as a greater fraction of the applied IrO*_x_* loading is electrochemically accessible at lowest loadings (*26*, *27*, *63*, *64*).

Alternatively, a common practice is to use electrochemical double-layer capacitance, which can act as a proportional approximation for ECSA when specific capacitance is constant, to normalize OER current (*28*, *30*, *31*). OER performance CVs of these IrO*_x_* samples standardized by electrochemical capacitance show excellent normalization, similar to results obtained when using quantity of Hg deposited/stripped as a normalization factor for these materials (Fig. 6C). In this case, the excellent normalization and consistency in results of capacitance-normalized OER activity across IrO*_x_* samples with various mass loadings is expected because all samples consist of identical IrO*_x_* catalyst (with identical specific capacitances). We note that for materials that experience notable reconstruction during extended testing, their specific capacitance will likely change over the course of testing such that comparisons made with capacitance-normalized OER activity will no longer be accurate, while the Hg UPD approach is expected to maintain accuracy. Both Hg UPD and double-layer capacitance measurements probe electrochemical surface area through similar fundamental processes, where ions in solution transport to the interface between electrode and electrolyte and build up either as a bound monolayer (Hg UPD) or as an electrochemical double layer (capacitance). Because of their similarities, when the electrode material and environment is kept constant (as we do within this work), we expect and observe capacitance values and the quantity of Hg deposited/stripped for electrodes to follow a similar relationship to catalyst mass loading (Fig. 6D). Therefore, with this set of samples, the ratio of capacitance to the quantity of Hg deposited/stripped remains very similar, with a slight decrease as IrO*_x_* loading increases (Fig. 6D, inset). However, when comparing more varied samples across literature or materials that undergo reconstruction during OER testing, capacitance normalization is prone to highlighting differences in the overall electrode composition, morphology, and environment, while Hg UPD will consistently only reflect changes in ECSI.

Particulate IrO*_x_* is used throughout this work to investigate the Hg UPD technique, but other related samples of particulate rutile IrO_2_ and Ir metal were also studied (figs. S14 and S15). Similar to mercury adsorption on IrO*_x_*, mercury absorption is observed to start occurring at 0.9 V_NHE_ for both IrO_2_ and Ir metal. The *I*_Hg_ and cumulative charge curves for both samples show good Hg deposition and stripping reversibility, but rutile IrO_2_ is observed to have highly symmetric adsorption and stripping features, with a Hg monolayer adsorption peak at 0.8 V_NHE_ and desorption peak centered at 0.85 V_NHE_. *I*_Hg_ and cumulative charge curves for the Ir metal–based electrode is observed to have asymmetrically shaped adsorption and stripping features with peaks that are broader and occur at more cathodic values than for the Ir oxide samples; the results for Ir metal reveal a broad Hg monolayer adsorption peak at 0.78 V_NHE_ and desorption peak centered at 0.82 V_NHE_.

## DISCUSSION

Here we have identified, carefully analyzed, and proposed a robust Hg UPD protocol for ECSI quantification on electrodes using a ubiquitous RRDE system. Development of this tool will bring unprecedented clarity to evaluate the relevant, accessible surface active sites for the vast range of Ir-based oxides that are broadly studied for the OER, particularly in acidic conditions as applicable to PEMWE technologies, as well as to a wide range of other electrochemical processes.

Hg deposition and stripping on Ir is a complex process that involves several electrochemical redox reactions and a chemical reproportionation reaction. We have identified two critical potential regions for Hg deposition and stripping: a regime over which monolayer-type coverage behavior on Ir sites is observed, and which bulk multilayers of Hg onto both Ir and C are expected. We have therefore proposed a Hg UPD protocol in the potential range of 1.2 to 0.7 V_NHE_, over which Hg deposition and stripping in a monolayer-like fashion is observed and analyzed as a measure of ECSI. The use of an RRDE with excellent current resolution is necessary to accurately deconvolute the competing redox and reproportionation reactions that cause excess reductive current to be observed at the disk over the Hg deposition and stripping regions of the CV. Once deconvoluted, the analyzed CVs of *I*_Hg_ show highly reversible Hg deposition and stripping from the electrode, corroborating conclusions from XPS characterization of the electrodes, as well as established understanding from EQCM results reported previously in the literature.

We characterize the nature of the Hg UPD CVs under various rotation rates and find that a less complete monolayer is expected at higher rotation rates, due to the increased flux of Hg^2+^ ions in solution, which cause increased rates of reproportionation of existing Hg^0^ at the electrode surface. We ultimately recommend a rotation rate of 400 rpm and provide a detailed electrochemical protocol for conducting Hg UPD for ECSI quantification.

When using our Hg UPD protocol and normalizing OER performance CVs of electrodes with various IrO*_x_* loadings by quantity of Hg deposited/stripped, we find that this metric is an excellent normalization factor and effectively collapses each electrode’s current density to one universal curve for IrO*_x_* to reveal and reflect the nature of this material’s intrinsic activity. Electrochemical double-layer capacitance similarly effectively normalizes OER performance of well-behaved catalyst materials like IrO*_x_*, when examined at short time scales as demonstrated here. However, we note the limitations of using electrochemical capacitance for assessing ECSA and active site density for many Ir-based oxides and complex electrode structures.

A Hg UPD quantification method offers key advantages over a traditional capacitance measurement and has the potential to enable highly quantitative comparison of varying materials’ ECSI and intrinsic activity measures. While electrochemical capacitance is often approximated as a proportional measure of an electrode’s electrochemical surface area, there are critical drawbacks to using it as a normalization factor (*29*). To meaningfully compare results across literature and convert a capacitance value into ECSA, a specific capacitance value is required, which is dependent on electrode composition, morphology, and electrolyte environment. However, specific capacitance values are infrequently measured, and when reporting does occur, these values can vary by two or three orders of magnitude. For example, a recent study determined specific capacitance values for their Ir oxide films in acidic electrolyte of ~1 mF/cm^2^ (*33*), while other studies commonly use a highly generalized value of 0.04 mF/cm^2^. The value of 0.04 mF/cm^2^ has regularly been applied to metal and metal oxide materials in acid despite its calculation from a limited set of reported specific capacitance measurements of Pt-, Ni-, Mo-, and Cu-based electrodes in H_2_SO_4_ solutions, for which the individual specific capacitance values themselves span an order of magnitude (*32*).

Additionally, measured capacitive current is often convoluted with current from other processes, including minor faradaic processes, ion adsorption, and corrosion (*17*, *34*–*37*). Electrochemical capacitance also fails to account for differences in active site (Ir) density on electrodes, with metal and metal oxide surfaces generally contributing to the final capacitance measurement. As researchers continue to explore materials with minimized Ir content, this poses a challenge in determining a reliable TOF based on Ir surface accessibility. We therefore alternatively propose and see great merit in the use of the Hg UPD protocol as a method to measure and quantify the quantity of OER-relevant ECSI on electrocatalysts for OER activity normalization. Considering the normalization tools currently at our disposal (especially for powder-based materials), we propose the Hg UPD protocol and its analysis is used for the normalization of OER performance to electrochemically accessible Ir sites on electrodes.

## MATERIALS AND METHODS

### Electrode fabrication

Electrodes were prepared for RRDE experiments using a Pine Research Instrumentation E6R2 Fixed-Disk RRDE tip. The RRDE tip consists of a 15.0–mm–outer diameter polytetrafluoroethylene (PTFE) shroud and a 5.5–mm–outer diameter fixed GC disk with an active electrode area of 0.238 cm^2^. The RRDE tip is equipped with a Pt ring with an outer diameter of 8.50 mm and inner diameter of 6.50, with a PTFE space of 0.5 mm between the disk and ring. The GC disk and Pt ring were always physically cleaned in between experiments and before drop-casting catalyst ink by physical polishing on a micro-cloth surface with a 0.05-μm suspended alumina solution. Polishing solution was subsequently rinsed from the RRDE tip, and the surface was wiped with a Kimwipe before drop-casting ink to ensure a clean surface. The Pt ring was additionally electrochemically cleaned before all experiments by performing rapid cyclic voltammetry at 200 mV/s in the range of 0.25 to 1.3 V_NHE_ until the voltammogram stabilized.

Catalyst ink was prepared by suspending commercial IrO*_x_* powder in a mixture of 97% by volume isopropyl alcohol and 3% Nafion 117 solution (Sigma-Aldrich, 5% in a mixture of lower aliphatic alcohols and water). The ratio of catalyst to solution was measured such that drop-casting 12.2 μl of ink onto the GC disk with a geometric area of 0.238 cm^2^ yielded a loading of 80 μg_Ir_/cm^2^. Similarly, a separate Vulcan carbon (FuelCellStore, Vulcan XC-72) ink was prepared with a ratio of 2 mg_C_/ml_ink_, with ink liquid volume consisting of 97% isopropyl alcohol and 3% Nafion 117 solution (Sigma-Aldrich, 5% in a mixture of lower aliphatic alcohols and water). A consistent quantity of 6.1 μl was deposited onto the GC working electrode after the IrO*_x_*-based ink dried for each sample. SEM images of electrodes with various IrO*_x_* loadings, with and without Vulcan carbon, are provided in the Supplementary Materials (fig. S16). The images show general even dispersion of IrO*_x_* on the GC disk substrate.

### Materials

Commercial IrO*_x_* (Alfa Aesar, Premion, 99.99% metals basis) was purchased and used as a benchmarking material for this work. The powder x-ray diffraction (PXRD) pattern of the commercial IrO*_x_* shows that a small amount of Ir metal is present (fig. S17). The IrO*_x_* powder is nanoparticulate (fig. S16) and is relatively high surface area, 39.1 m^2^/g, as determined from a N_2_ isotherm and BET surface area analysis. BET surface area analysis was conducted at Northwestern’s Reactor Engineering and Catalyst Testing (REACT) center.

Additional rutile IrO_2_ and Ir metal–based electrodes were prepared with 40 μg_Ir_/cm^2^ and Vulcan carbon in an identical manner to the IrO*_x_* electrodes. Ir metal powder (~325 mesh, 99.9%) was used as purchased from Alfa Aesar. IrO_2_ powder was synthesized by calcining the commercial IrO*_x_* (same as that used throughout this work) powder in an alumina boat in a box furnace in air at 450°C for 3 hours. Rutile phase crystallinity was confirmed via PXRD.

### Electrochemical testing

All electrochemical measurements were conducted in a beaker with 30 ml of 0.1 M HClO_4_ (pH 1.1) aqueous electrolyte prepared with Milli-Q water. A clean Pt wire counter electrode and Ag/AgCl (saturated KCl) reference electrode were used with the RRDE and measured in bi-potentiostat mode using a VSP-3 Biologic potentiostat. The Ag/AgCl reference was calibrated in electrolyte versus the reversible hydrogen electrode (RHE) before electrochemical measurements and found to have a calibration value of 0.265 V_RHE_. To determine the calibrated value against the NHE, we used the calibrated value of the reference electrode against RHE in 0.1 M HClO_4_ and subtracted 59 mV per pH unit [0.265 − (0.059*1.1)] to determine a calibrated value of 0.200 V_NHE_.

To prepare electrolyte for mercury UPD testing, 0.612 ml of mercury nitrate solution [0.05 M Hg(NO_3_)_2_, “Fluka” Honeywell] was added to 30 ml of 0.1 M HClO_4_ electrolyte and the RRDE was left to rotate in the solution for approximately 10 s to allow the electrolyte solution to become homogeneous before testing.

Electrochemical capacitance of electrodes in this work was determined through a traditional variable scan rate method in the capacitive portion of a CV. We used a voltage range of 0.8 to 1.0 V_Ag/AgCl_ and selected scan rates of 5, 10, 25, 50, and 100 mV/s and observed the positive and negative current value at 0.9 V_Ag/AgCl_ (fig. S10A). The absolute value of current at 0.9 V_Ag/AgCl_ was plotted against scan rate, and a linear regression was conducted to obtain a slope, the average value of which (from the cathodic and anodic current curves) is our measured capacitance (fig. S10B). For electrodes with very high loadings of IrO*_x_* (160 μg_Ir_/cm^2^), data points at 100 mV/s were excluded as they deviated notably from the expected linear behavior versus scan rate. Capacitance raw data and analysis from a characteristic electrode with 40 μg_Ir_/cm^2^ and Vulcan carbon are reported in the Supplementary Materials (fig. S10).

### Materials characterization

XPS was conducted using a Thermo Fisher Scientific ESCALAB 250Xi with a monochromatic Al Kɑ emission source and a 500-μm spot size. Thermo Fisher Scientific Avantage software was used to fit all spectra. A Shirley background and Gaussian peaks were used to fit each spectrum. The position of the high-intensity C-F 1s peak was calibrated against the Au 4f 7/2 peak (set to 84.00 eV) using a sample of IrO*_x_* ink dried on a metallic gold substrate. The position of the C-F 1s peak was then shifted for each sample accordingly to charge shift each sample’s spectra. Scanning electron microscopy (SEM) was conducted using a Hitachi SU8030. A 10-kV accelerating voltage was used for all SEM imaging.
