# Supplementary material for: Quantification of electrochemically accessible iridium oxide surface area with mercury underpotential deposition
Source: Sci Adv. 2024 Nov 6;10(45):eadp8911. doi: 10.1126/sciadv.adp8911 (PMC11540024; doi:10.1126/sciadv.adp8911)
Supplement: Supplementary file 1 — Supplementary Text Figs. S1 to S17 Tables S1 to S5 [file sciadv.adp8911_sm.pdf]

Science Advances
AAAS

Supplementary Materials for

Quantification of electrochemically accessible iridium oxide surface area with mercury underpotential deposition

Jane Edgington *et al.*

Corresponding author: Linsey C. Seitz, linsey.seitz@northwestern.edu

Sci. Adv. **10**, eadp8911 (2024)
DOI: 10.1126/sciadv.adp8911

This PDF file includes:

Supplementary Text
Figs. S1 to S17
Tables S1 to S5

Supplementary Text

EC-lab (Potentiostat Control Software) Protocol
The following describes the final electrochemical protocol for performing Hg UPD/stripping experiments for Ir site quantification:

1. Pt ring clean (0.1 M $HClO_4$ always)
    a. CV 1.1 V_{NHE} – 0.25 V_{NHE}

The following steps are completed in 0.1 M $HClO_4$, then with 0.1 M $HClO_4$ + 1 mM $Hg(NO_3)_2$ under a rotation rate of 400 rpm. All ring current measurements are collected with an optimized bandwidth of 3. Disk current measurements are collected with a bandwidth of 5 (EC-Lab setting):

2. CA – RCA
    a. Disk Voltage: Hold @ 1.2 V_{NHE} until stable (~30 s – 1 min)
    b. Ring voltage: 1.3 V_{NHE}
    c. *Notes: This step allows the ring voltage to reach a stable baseline before starting the Hg UPD protocol, which improves reproducibility of CVs between samples.*
3. CV – RCA
    a. Disk Voltage: CV at 5 mV/s from 1.2 V_{NHE} to 0.7 V_{NHE}, repeat at least once and end at 0.7 V_{NHE}.
    b. Ring voltage: 1.3 V_{NHE}
    c. *Notes: These CV cycles are the cycles shown for Hg UPD analysis. The second cycle is always used for the final Hg UPD analysis when Hg is in electrolyte. The background CV used in analysis is typically the 3rd or 4th CV, depending on how long it takes for the CV to stabilize. If OER has previously been conducted with the sample, typically several cycles are needed to remove the excess adsorbed oxygen species on the surface to yield a stable baseline CV.*
4. CA – RCA
    a. Disk Voltage: Hold @ 0.7 V_{NHE} for 2 min
    b. Ring Voltage: 1.3 V_{NHE}
    c. *Notes: The current response from the disk and ring during this step is used to calculate the collection efficiency (N) of the ring for a direct-measurement N determination.*
5. CV – RCA
    a. Disk Voltage: CV at 5 mV/s from 0.7 V_{NHE} – 1.0 V_{NHE}, 5 mV/s
    b. Ring Voltage: 1.3 V_{NHE}
    c. *Notes: This step is not used in any formal analysis but is incorporated to strip the Hg from the electrode surface after the 2 min hold at 0.7 V_{NHE}.*

A schematic of this protocol, and characteristic data is shown in Fig. S6.

Ring Collection Efficiency

Ferrocyanide/ferricyanide ($Fe(CN)_6^{3-} + e^- \rightarrow Fe(CN)_6^{4-}$) was used to measured ring and disk current to determine the collection efficiency of the Pt ring (N) with a clean GC disk head under various rotation rates. An average N of 0.382 is determined for these experiments.

The large gap between the baseline values at 1.0-1.2 V_{NHE} (Fig. S2.D) when using the N value of 0.382 indicates a high degree of irreversibility of the Hg UPD system, where the calculated quantity of Hg deposited is far greater than that stripped. This is a mathematical artifact from using an inappropriate N of 0.382. Cumulative charge curves using the N values found through the direct measurement protocol during an Hg UPD protocol show excellent reversibility.

Additional experiments were carried out to determine the collection efficiency of the Pt ring with a glassy carbon disk with 40 $\mu g_{Ir}/cm^2$ IrO_x and Vulcan carbon deposited. Ferrocyanide ($[Fe(CN)_6]^{3-}$) was used to measured ring and disk current to determine the collection efficiency under various rotation rates. N results are shown to vary in these experiments more than the clean GC system.

Monolayer Occupancy & Conversion Factor Estimation

For the 40 $\mu g_{Ir}/cm^2$ IrO_x sample shown in Fig. 5 of the main article, monolayer occupancy is estimated from BET surface area of the IrO_x powder, as well as an average areal surface density estimation of 5.99 atoms Ir per nm^2 from the [100] and [110] facets of rutile IrO_2, as reported from Ma, Zhong, et al. and A. A. Bolzan, et al.*(61,62)* The monolayer occupancy is calculated with the assumption that a Hg monolayer deposits in a 1:1 ratio to Ir. We note that IrO_x is expected to be less dense than crystalline rutile IrO_x, and therefore IrOx surface area reported in Table S3 is overestimated. Additionally, we expect a sizeable fraction of the deposited powder IrO_x surface area on the electrode to be inaccessible to Hg UPD, due to powder packing on the substrate, adding to the overestimation of IrO_x surface area.

The conversion factors of 85 and 62 $\mu C_{Hg}/nmol_{SurfaceIr}$ are calculated using an average areal surface density (or planar density) estimation of 5.99 atoms Ir per nm^2 (as specified above) and 13.4 atoms Ir per nm^2, assuming a surface facet of [100] of Ir metal, with a Ir atomic radius of 0.1365 nm.

Alternative Ir-Hg Quantification and Mathematical Approaches

The quantification of Hg^+ is critical in carrying out the mathematical analysis to determine the charge associated with Hg-Ir monolayer deposition and stripping. While there is no distinct feature in the ring current to that mirrors the anodic Hg stripping feature, there is a notable amount of Hg+ production during the anodic sweep of the CV that interferes with the baseline of the Hg stripping peak (Fig. S7.A). While in theory the charge associated with the Hg-Ir stripping feature in the disk current CV could solely be used to estimate ECSI (electrochemically accessible surface Ir sites), the interference from Hg^+ production renders the practice of determining solely the charge associated with the Hg-Ir stripping process highly mathematically ambiguous and error-prone. For example, in this case we cannot simply use the difference between $I_{disk,Hg}$ and $I_{disk,0.1MHClO4}$ to calculate the Ir-Hg stripping charge, as it would clearly give a nonphysical result and fail to capture the entirety of the Ir-Hg stripping peak (Fig. S7. A). This nonphysicality is also illustrated clearly in Fig. S7.B, when comparing the background-subtracted I_{Hg} CV curves. Here we can define I_{Hg} as we do in the main text and account for Hg^+ production (green curve), or we can define I_{Hg} without Hg^+ consideration (blue curve). We clearly observe that integration of

the Ir-Hg stripping feature most accurately captures the reversible stripping feature when Hg^+ formation is accounted for (per our protocol recommendation).

Another approach might be to draw an alternative linear baseline on the Hg-added CV and integrate to find the stripping peak charge. However, the drawing of that baseline is mathematically ambiguous and up to the discretion of the researcher, as different interpretations of an "appropriate" baseline are nearly guaranteed (Fig. S7.C). Any linear baseline is also fundamentally nonphysical, as we know that the true appropriate baseline subtraction is a combination of the background non-Hg CV ($I_{disk,0.1MHClO4}$) and the current associated with Hg^+ production ($I_{ring,Hg}$), both of which are nonlinear functions.

To explore an alternative approach in an effort to possibly offer a simpler calculation for researchers to calculate an ECSI estimate, we explored the scenario of no rotation (and therefore no Hg^+ ring detection). Under a no rotation condition, we observed better agreement between the baseline of the Hg-added and background 0.1 M $HClO_4$ CV around the stripping feature and more reversible charge behavior with deposition and stripping (Fig. S8.A). This integrated feature is more mathematically plausible than that for the 400 rpm scenario when using $I_{disk,0.1MHClO4}$ as a baseline (i.e. defining $I_{Hg} = I_{disk,Hg} - I_{disk,0.1MHClO4}$). Additionally, this simplified protocol would enable the application to alternative electrode geometries without the constraints of an RRDE. However, the relationship between this peak integration value at 0 rpm versus mass loading on the electrode is not consistent with that observed for the results of our recommended protocol at 400 rpm nor electrode double layer capacitance. We can observe that both capacitance and our cumulative Hg dep/strip charge value at 400 rpm are similarly excellent normalization factors for OER activity of electrodes with various mass loadings (Fig. S8.D). However, when using the integration of the stripping peak at 0 rpm (and not accounting for Hg^+ production) it appears this method is disproportionately affected by mass transfer limitations and underestimates ECSI for high loading samples (compared to estimates for lower loading samples). This ECSI underestimation for high samples is seen in the large decrease in ratio of 0 rpm Hg results to capacitance (Fig. S8.C) for samples with greater mass loadings. This ECSI underestimation from a 0 rpm Hg UPD condition can lead to inconsistent site and OER performance normalization. Because of this, we maintain that the originally recommended protocol under rotation at 400 rpm with use of an RRDE for Hg^+ probing produces the best, most robust and reliable ECSI estimation and OER normalization factor.

<u>Hg UPD with / without Vulcan Carbon</u>

Hg UPD experiments and OER activity normalizations were completed for 40 $\mu g_{Ir}/cm^2$ samples prepared with and without the Vulcan carbon support. We note that the addition of Vulcan carbon to the electrode also improves the electrode's ability to return to baseline current on the anodic sweep of the CV during Hg UPD, seen from the zero-magnitude current anodic of 1.0 V_{NHE} on the anodic sweep for the sample with added Vulcan carbon.

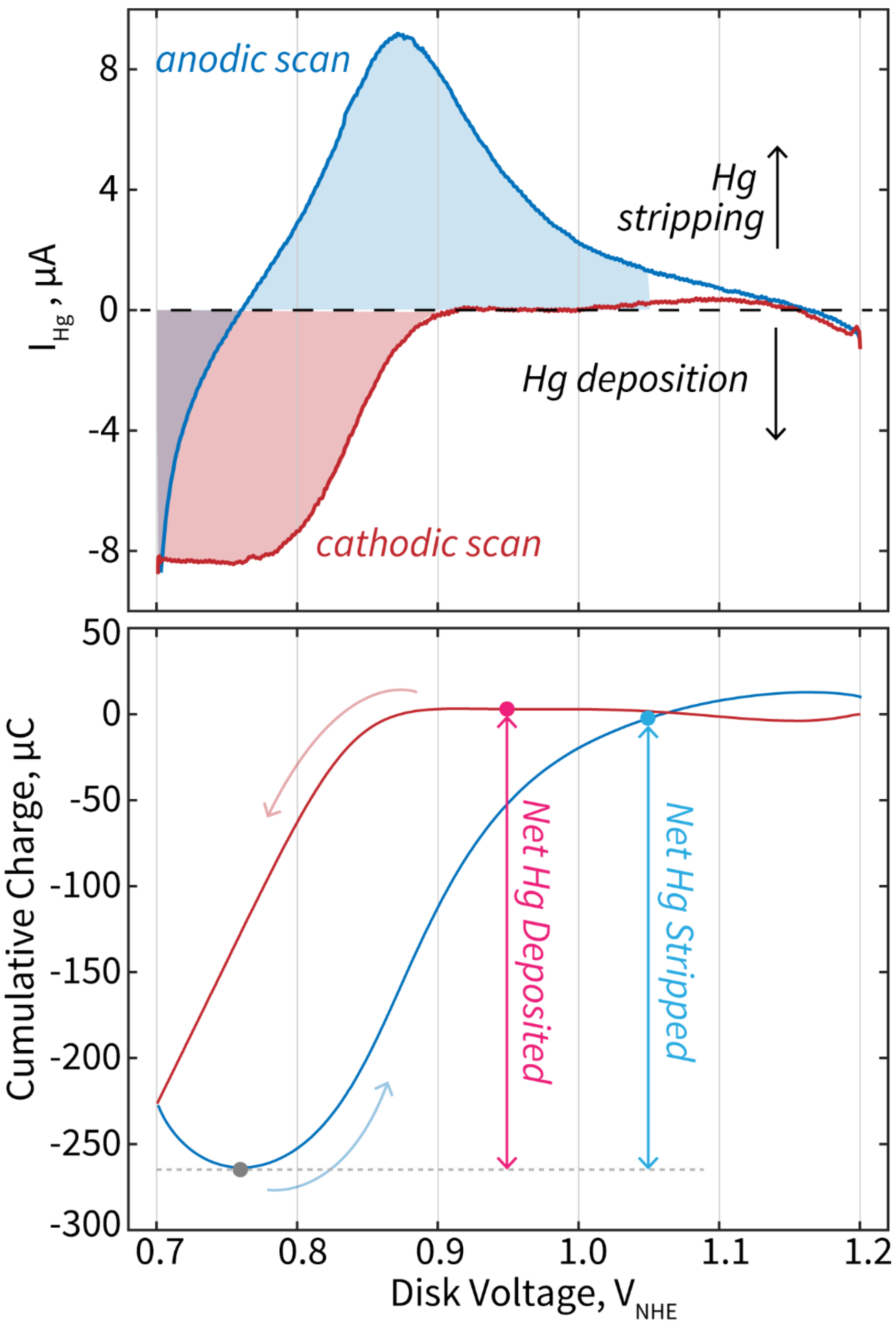

Fig. S1.

I_{Hg} and Cumulative charge from Hg deposition and stripping during a Hg UPD CV. Data shown is for a 40 $\mu g_{Ir}/cm^2$ IrO_x sample with Vulcan carbon, in 0.1 M$HClO_4$ + 1 mM $Hg(NO_3)_2$ electrolyte rotated at 400 rpm. Arrows indicate the sweep direction of the CV, cathodic to anodic. The circles on the plot indicate the integration bounds from which the net amount of Hg deposited and stripped are calculated (0.95 V_{NHE} – 1.05 V_{NHE})

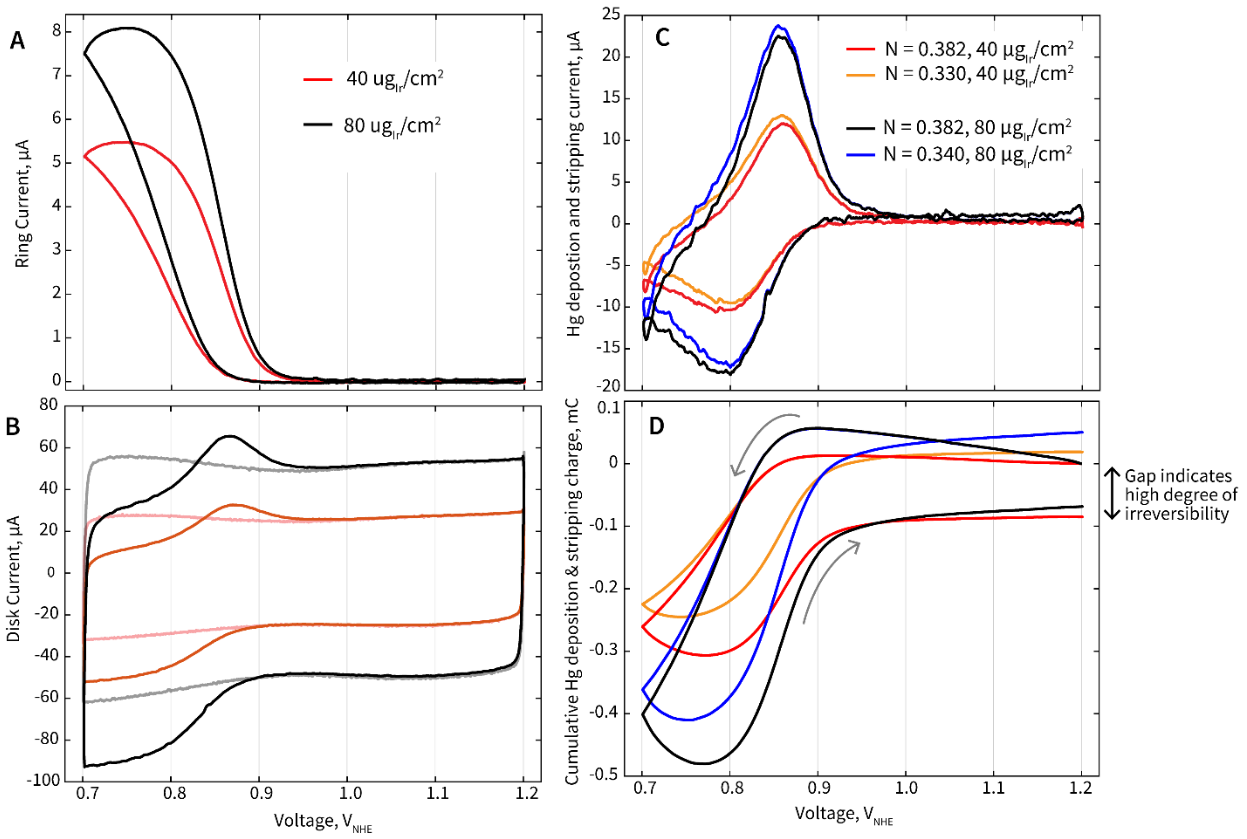

Fig. S2.

Electrochemical data and analysis for GC/Vulcan/IrO_x electrodes following the recommended Hg UPD CV protocol with various N. A. Ring current vs. disk voltage for samples with 40 and 80 $\mu g_{Ir}/cm^2$ IrOx and Vulcan carbon during a Hg UPD CV with 400 rpm in 0.1 M $HClO_4$ + 1 mM $Hg(NO_3)_2$. **B.** Disk current vs. disk voltage during a Hg UPD CV, shown for 0.1 M $HClO_4$ + 1 mM $Hg(NO_3)_2$ and for background CVs collected in 0.1 $HClO_4$ (lighter lines). Legend of A applies to B. **C.** Analyzed Hg deposition and stripping current vs. disk voltage, using CE values listed. The N value of 0.382 is determined from the ferrocyanide/ferricyanide redox with a clean GC disk, while the other N values of 0.33 and 0.34 were determined from the direct measurement of N during the Hg UPD protocol. **D.** Cumulative charge associated with Hg deposition and stripping throughout the CV, using N values listed. Legend of C applies to D.

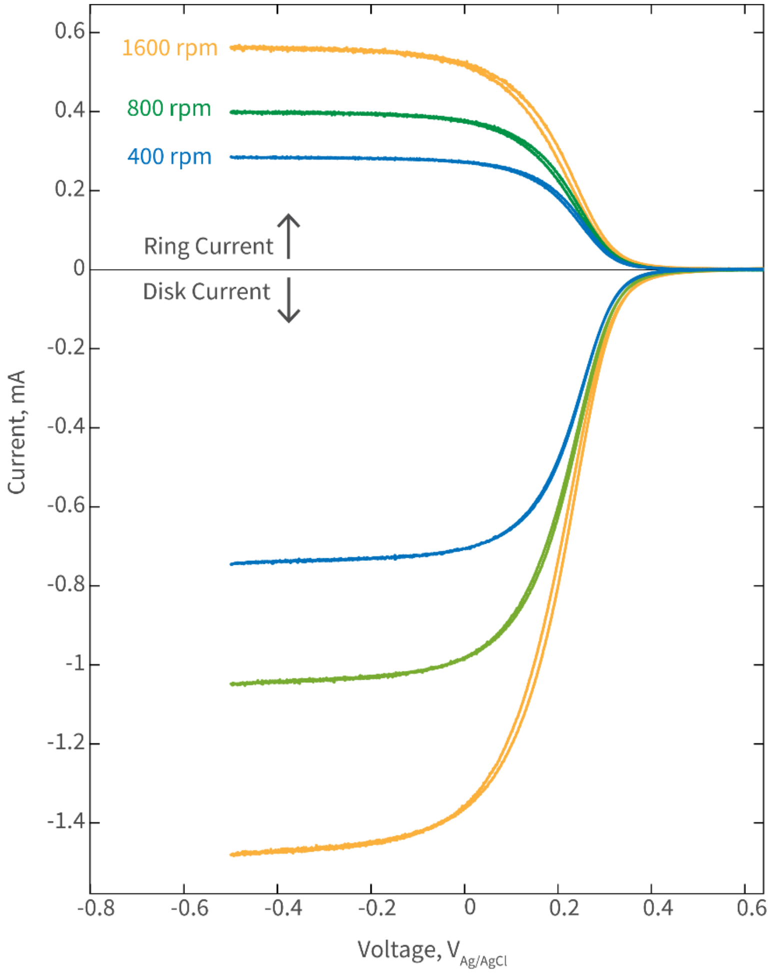

Fig. S3.

Ideal case ring and disk current for N determination. GC disk (clean, no ink deposited) and Pt ring current vs. disk voltage for various rotation rates in 1.0 M KNO_3 / 0.01 M $K_3[Fe(CN)_6]$.

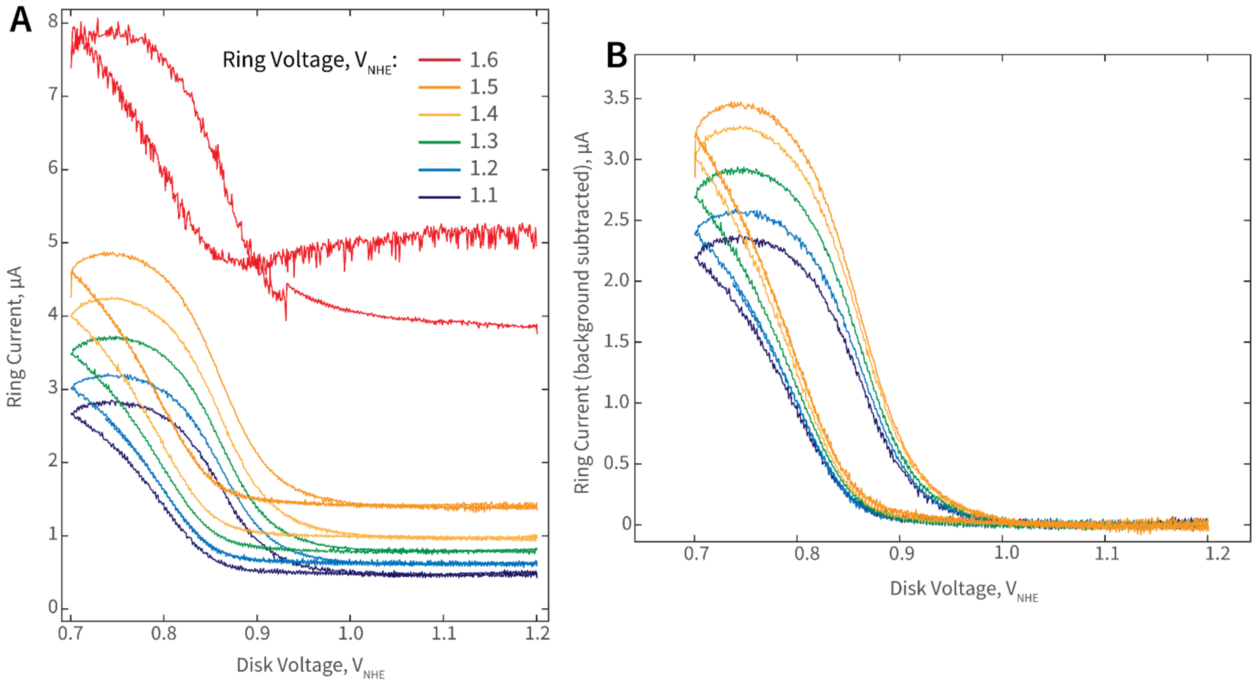

Fig. S4.

Ring sensitivity studies to Hg^+. A. Ring current (unprocessed) vs. disk voltage during CVs in 0.1 M$HClO_4$ + 1 mM $Hg(NO_3)_2$, with varying ring potentials at 800 rpm. The disk has an IrO_x loading of 40 $\mu g_{Ir}/cm^2$. **B.** Background-subtracted ring current vs. disk current from (A), excluding the 1.6 V_{NHE} data, which could not be effectively background subtracted given the electrode's instability under such an oxidizing potential. Legend of A applies to B.

.

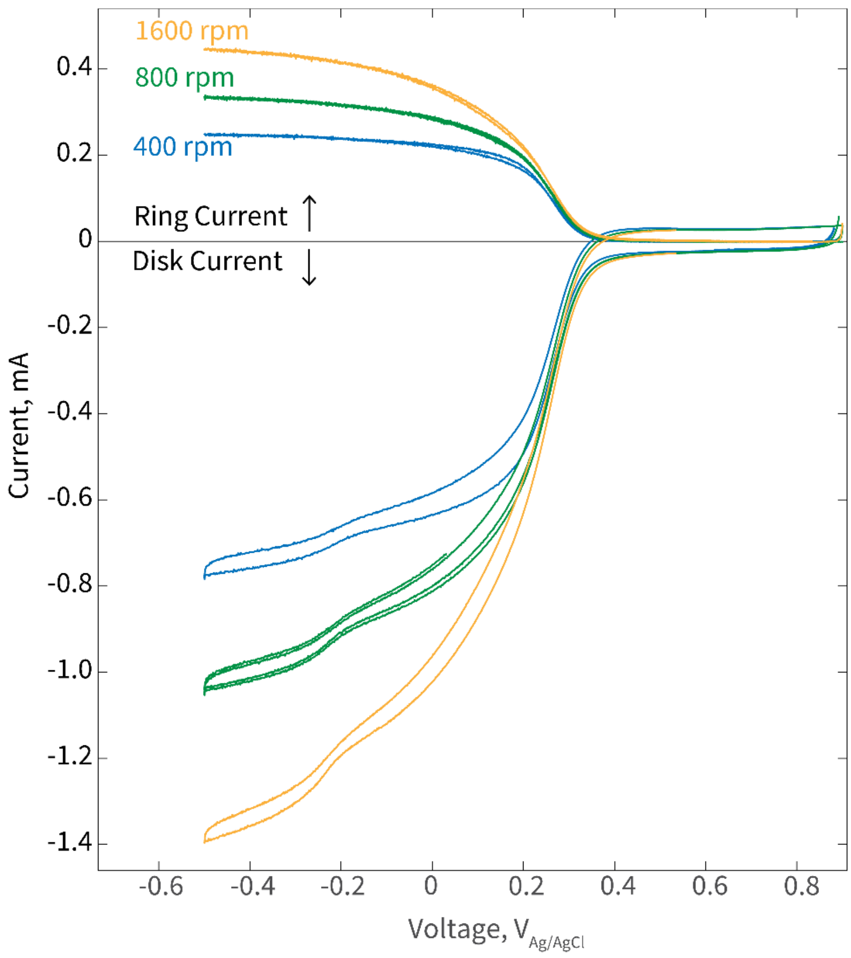

Fig. S5.

Actual use case ring and disk current for N determination. Glassy carbon disk with 40 $\mu g_{Ir}/cm^2$ IrO_x and Vulcan carbon deposited and Pt ring current vs. disk voltage for various rotation rate in 1.0 M KNO_3 / 0.01 M $K_3[Fe(CN)_6]$.

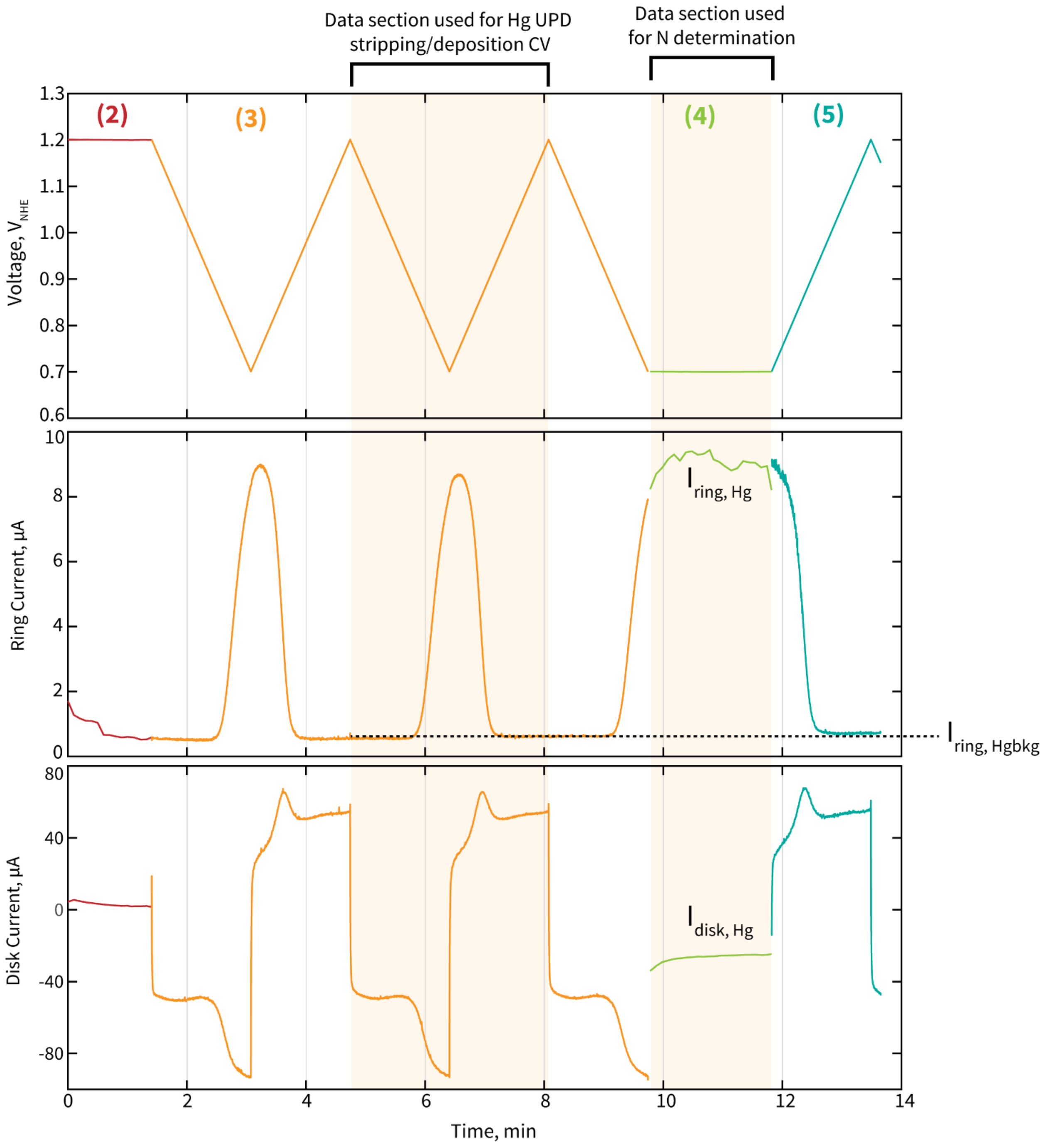

Fig. S6.

Recommended electrochemical protocol schematic and description/identification of the critical variables needed from the electrochemical data for formal Hg UPD analysis. Voltage, ring current, and disk current vs. time throughout a typical Hg UPD experiment, shown for 80 $\mu g_{Ir}/cm^2$ IrO_x sample with Vulcan carbon, in 0.1 M$HClO_4$ + 1 mM $Hg(NO_3)_2$ electrolyte, rotated at 400 rpm. The sections of the protocol that are used for formal data analysis are labeled above each of the shaded regions of the plot, as well as the protocol steps mentioned prior.

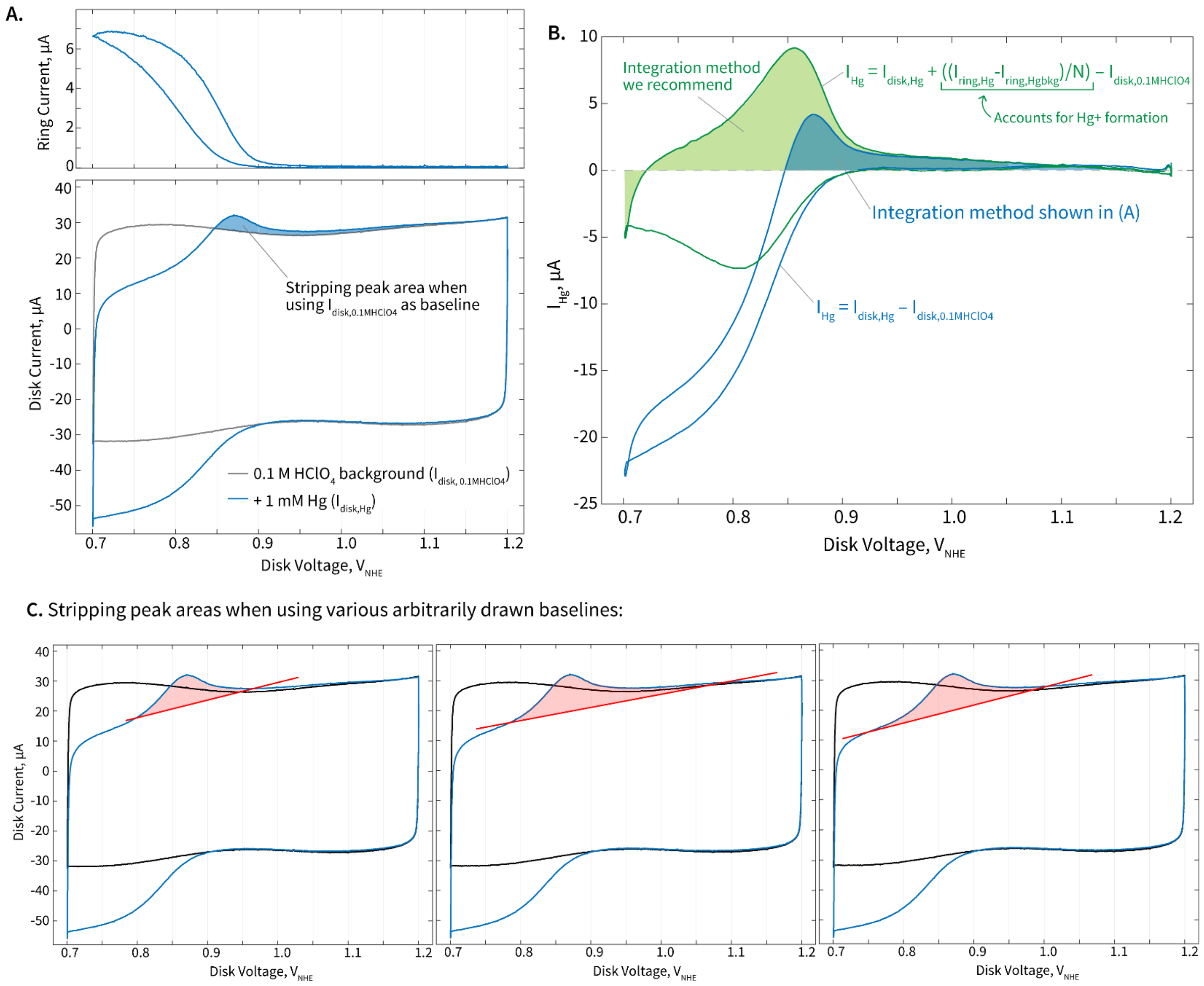

Fig. S7.

Visualization and comparison of alternative Hg UPD mathematical analyses. A. Ring current and disk current versus disk voltage conducted with a disk electrode consisting of a glassy carbon disk with Vulcan carbon and 40 $\mu g_{Ir}/cm^2$ IrO_x (GC/Vulcan/IrO_x) in 0.1 M $HClO_4$ with (and without) 1 mM $Hg(NO_3)_2$. **B.** I_{Hg}, interpreted two ways using data from (A). Green curve shows I_{Hg} as defined in the main body text, while blue curve shows I_{Hg} when failing to account for Hg^+ production and ring current. Integrated charge areas for the anodic stripping peak are shown in shaded areas with respective colors. **C**. Disk data from (A) with various, plausible linear baselines that may be selected if an appropriate baseline is not measured as shown in the green curve in (B). The respective stripping peak areas reflect the variation in peak area magnitude depending on the selected baseline.

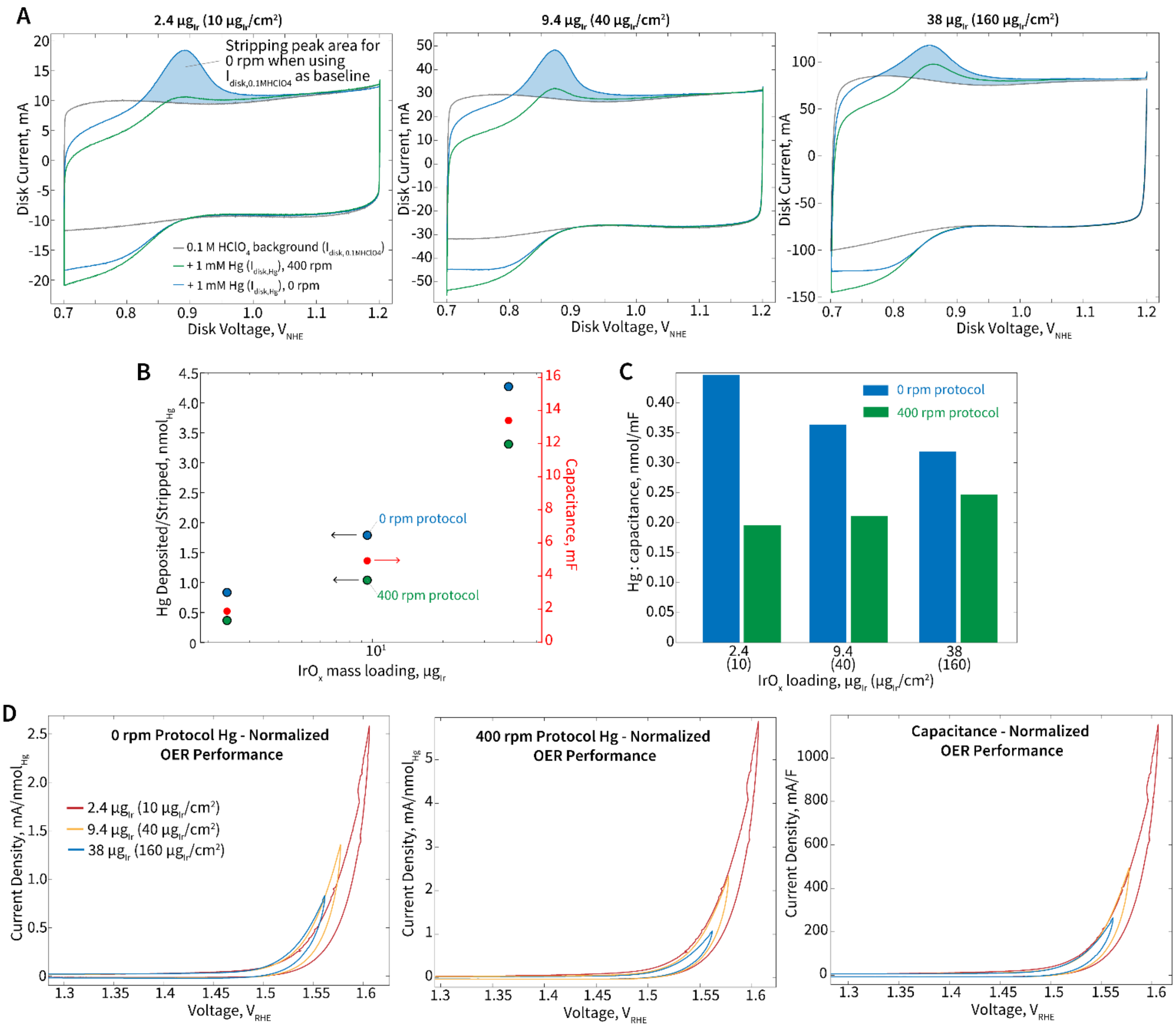

Fig. S8.

Comparison of alternative Hg UPD protocol at 0 rpm to the recommended 400 rpm protocol. **A.** Disk current versus disk voltage at 0 and 400 rpm conducted with a disk electrode consisting of a glassy carbon disk with Vulcan carbon and 10, 40, and 160 $\mu g_{Ir}/cm^2$ IrO_x (GC/Vulcan/IrO_x) in 0.1 M $HClO_4$ with (and without) 1 mM $Hg(NO_3)_2$. Shaded areas show the stripping peak area for the 0 rpm scenario when using the non-Hg 0.1 M $HClO_4$ CV ($I_{disk,0.1MHClO4}$) as integration baseline. **B.** Charge associated with Hg deposition and stripping (for 0 rpm case this only includes charge with Hg stripping, as calculated as shaded areas in A) for 0 and 400 rpm scenarios, and double layer capacitance for electrodes with 10, 40, and 160 $\mu g_{Ir}/cm^2$ IrO_x. 0 rpm protocol results are simply the integration of $I_{disk,Hg}$ with $I_{disk,0.1MHClO4}$ as a baseline, while the 400 rpm protocol involves the full recommended Hg UPD analysis provided in the main text. **C.** Ratios of Hg deposited/stripped to capacitance for 0 and 400 rpm scenarios. **D.** Normalized OER performance curves for electrodes with 10, 40, and 160 $\mu g_{Ir}/cm^2$ IrO_x when using results from 0 rpm Hg, 400 rpm Hg, and capacitance values as normalization factors.

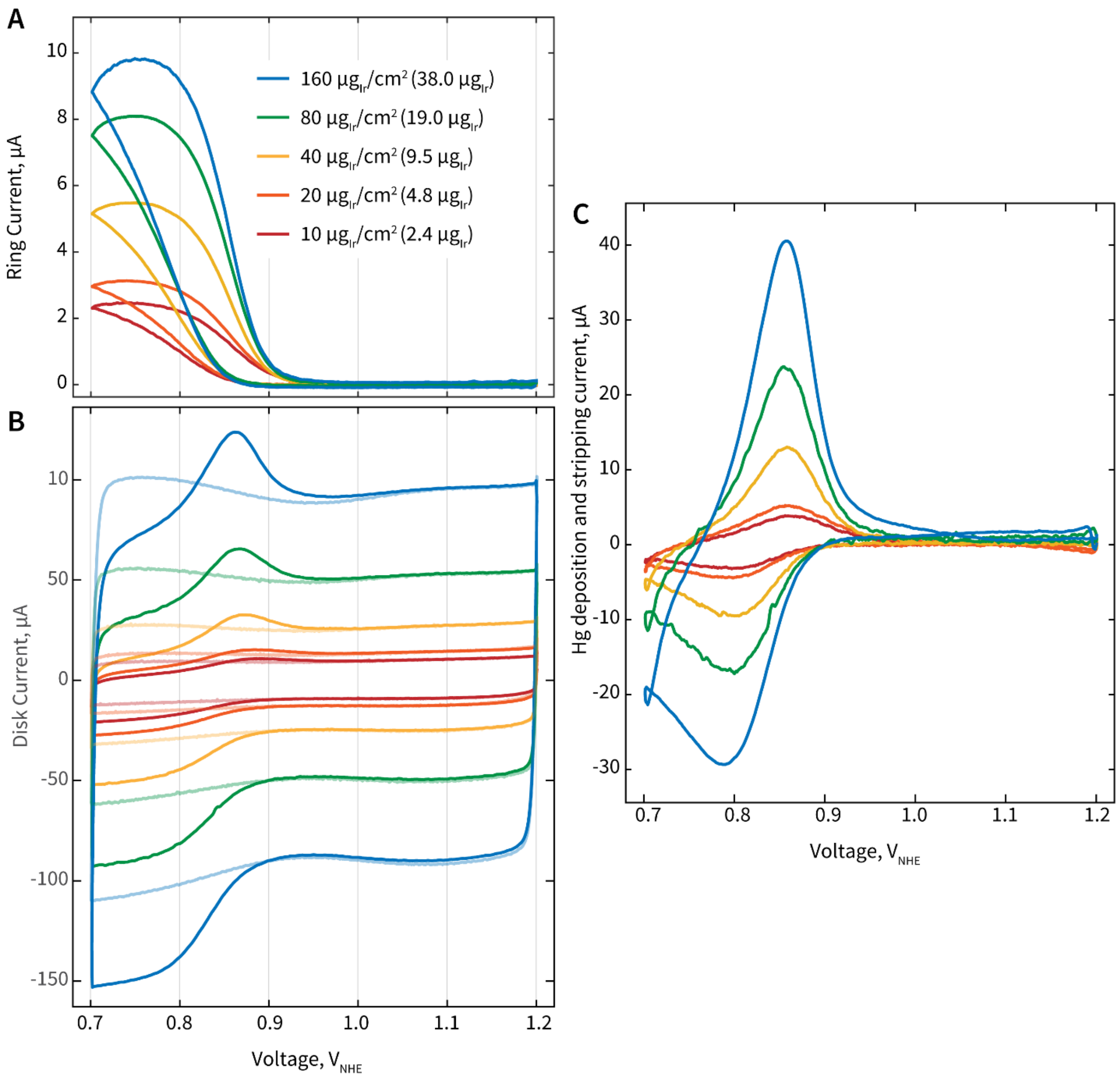

Fig. S9.

Additional electrochemical Hg UPD data for GC/Vulcan/IrO_x electrodes with varying IrO_x mass loading. A. Ring current vs. disk voltage during a Hg UPD CV at 400 rpm in 0.1 M $HClO_4$ + 1 mM $Hg(NO_3)_2$ with IrO_x loadings at the disk of 40-160 $\mu g_{Ir}/cm^2$, with μg_{Ir} specific loadings given in parentheses. **B.** Disk current vs. disk voltage during Hg UPD CVs, shown for 0.1 M $HClO_4$ + 1 mM $Hg(NO_3)_2$ and for background CVs collected in 0.1 $HClO_4$ (lighter lines). **C.** Analyzed Hg deposition and stripping current vs. disk voltage. Legend of A applies to B and C.

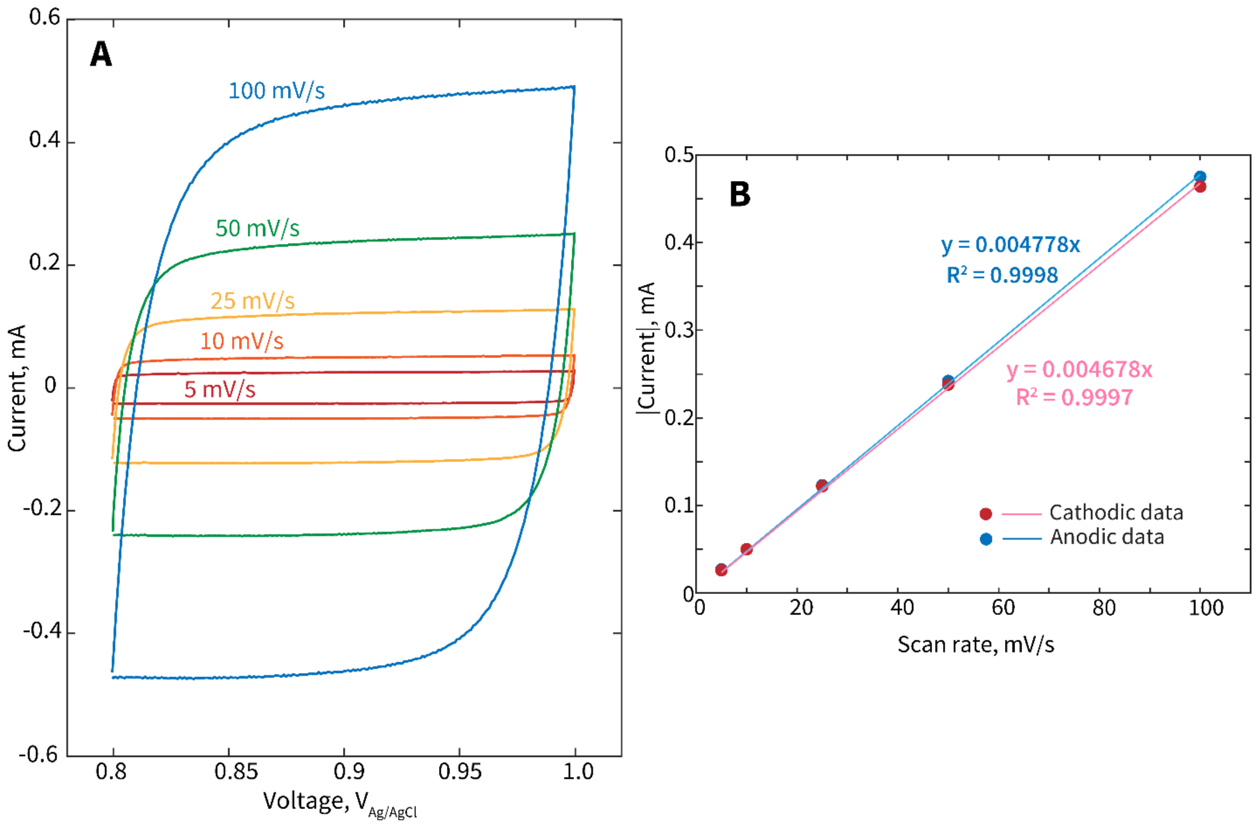

Fig. S10.

Characteristic electrochemical double layer capacitance data for a GC/Vulcan/IrO_x electrode. A. Capacitive current measurements for various scan rates of 5, 10, 25, 50, and 100 mV/s for an electrode with 40 $\mu g_{Ir}/cm^2$ IrO_x and Vulcan carbon in 0.1 M $HClO_4$. **B.** Absolute value of capacitive current as a function of scan rate, with linear regression fits (with y-intercept forced through zero), equations, and R^2 values. The ultimate capacitance value is an average of the cathodic and anodic linear regression slopes (slopes reported in units of Farads).

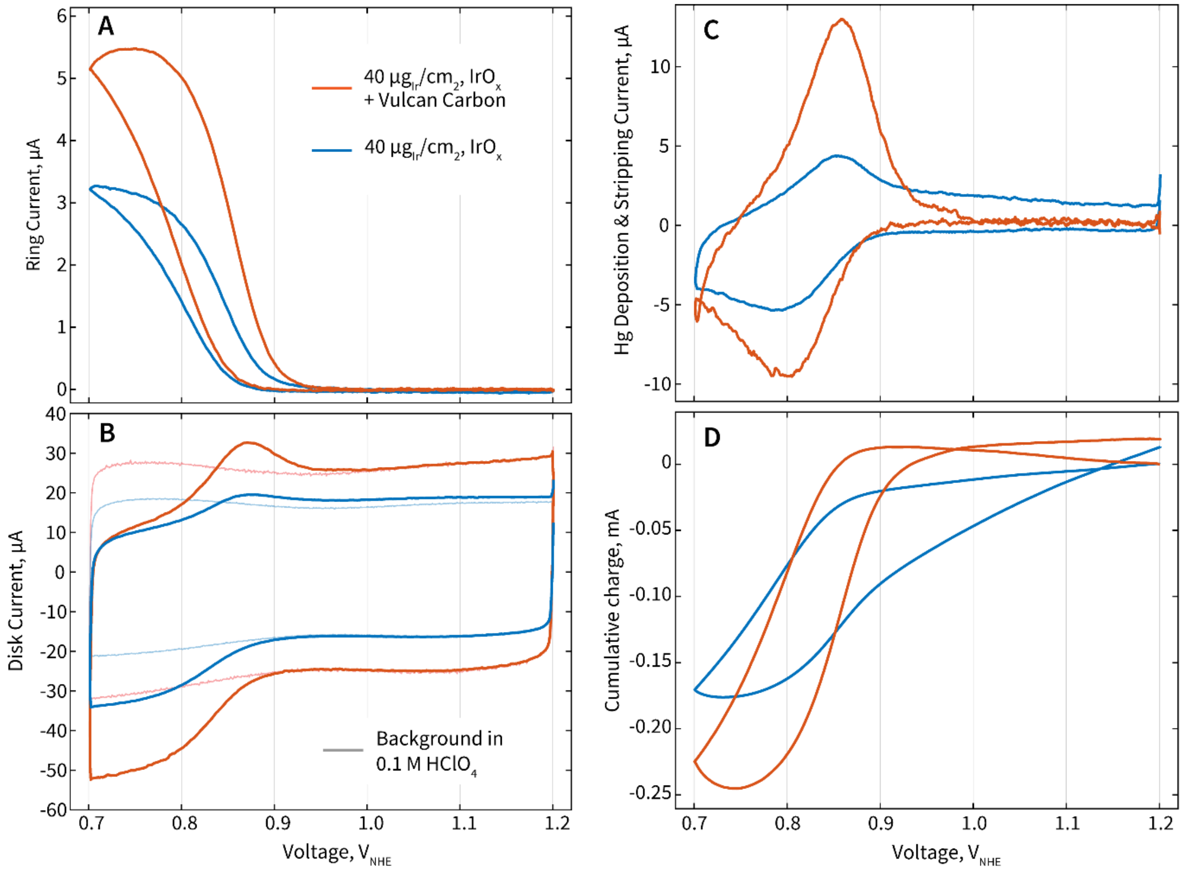

Fig. S11.

Electrochemical data and analysis for GC/Vulcan/IrO$_x$ and GC/IrO$_x$ electrodes. A. Ring current vs. disk voltage during a Hg UPD CV with 400 rpm in 0.1 M $HClO_4$ + 1 mM $Hg(NO_3)_2$. **B.** Disk current vs. disk voltage during a Hg UPD CV, shown for 0.1 M $HClO_4$ + 1 mM $Hg(NO_3)_2$ and for background CVs collected in 0.1 $HClO_4$ (lighter lines). **C.** Analyzed Hg deposition and stripping current vs. disk voltage **D.** Cumulative charge associated with Hg deposition and stripping throughout the CV. Legend of A applies to A-D.

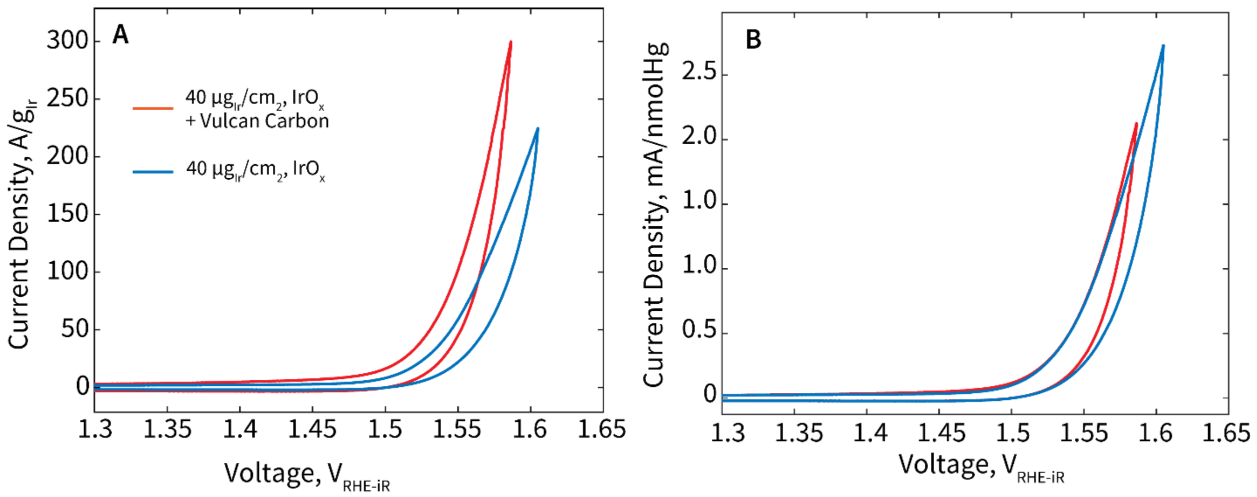

Fig. S12.

OER performance CVs for GC/Vulcan/IrO_x and GC/IrO_x electrodes with mass and Hg UPD-normalization. Curves collected under a rotation rate of 1600 rpm in 0.1 M$HClO_4$ show **A.** Mass-normalized current density vs. voltage and **B.** Hg UPD charge-normalized current density vs. voltage. Legend of A applies to B.

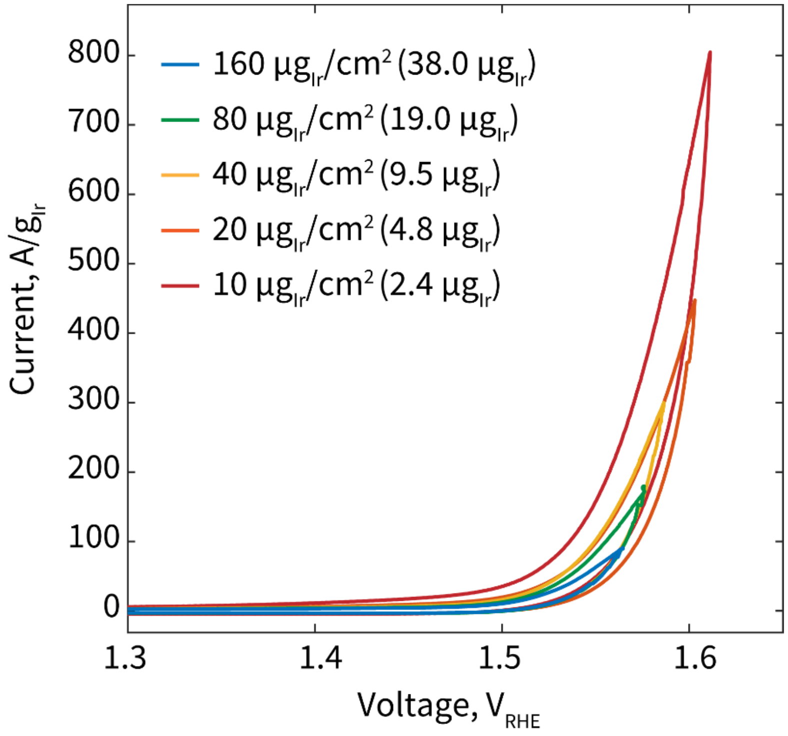

Fig. S13.

Mass-normalized OER performance CVs of electrodes with various mass loadings of IrO_x. All samples have a constant amount of Vulcan carbon applied and are measured in 0.1 $MHClO_4$ with 1600 rpm,. Voltage is iR compensated based on the series resistance at OCV of each sample.

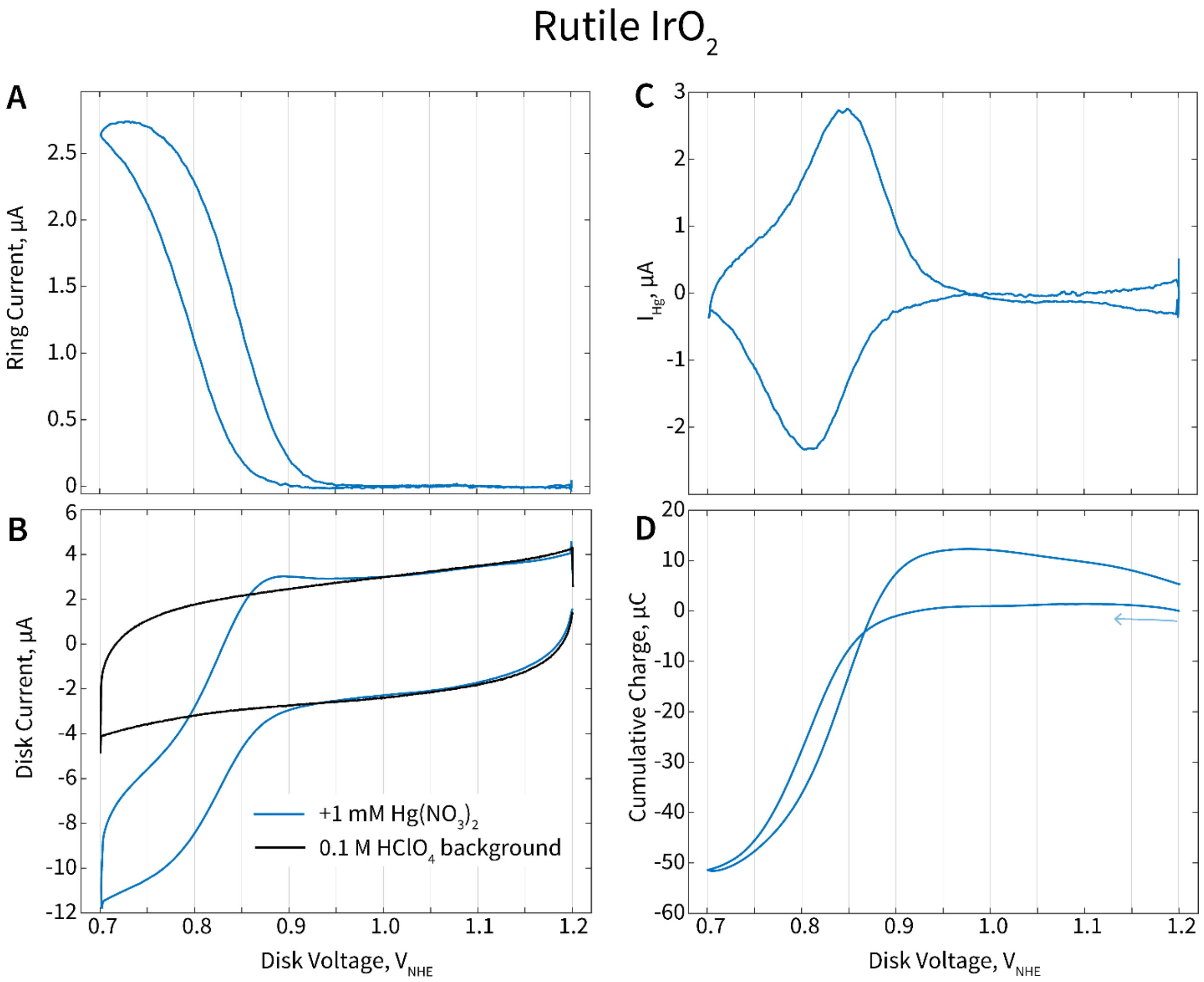

Fig S14.

Hg UPD experiments for 40 $\mu g_{Ir}/cm^2$ rutile IrO_2 with Vulcan carbon. A. Ring current vs. disk voltage during a Hg UPD CV with 400 rpm in 0.1 M $HClO_4$ + 1 mM $Hg(NO_3)_2$. **B.** Disk current vs. disk voltage during a Hg UPD CV, shown for 0.1 M $HClO_4$ + 1 mM $Hg(NO_3)_2$ and for a background CV collected in 0.1 $HClO_4$ (black line). **C.** Analyzed Hg deposition and stripping current vs. disk voltage **D.** Cumulative charge associated with Hg deposition and stripping throughout the CV. Light blue arrow indicates start of CV.

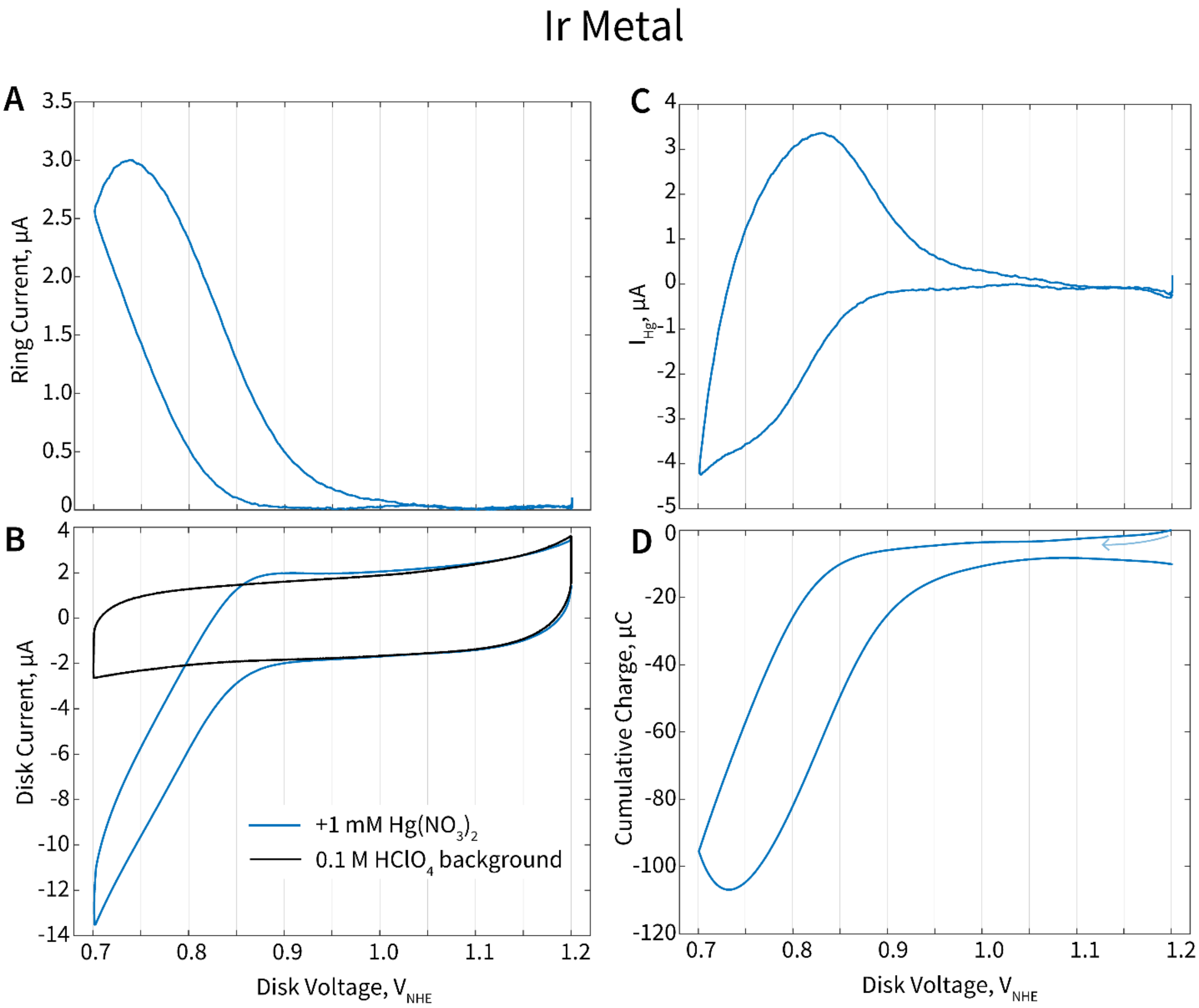

Fig. S15.

Hg UPD experiments for 40 $\mu g_{Ir}/cm^2$ Ir metal with Vulcan carbon. A. Ring current vs. disk voltage during a Hg UPD CV with 400 rpm in 0.1 M $HClO_4$ + 1 mM $Hg(NO_3)_2$. **B.** Disk current vs. disk voltage during a Hg UPD CV, shown for 0.1 M $HClO_4$ + 1 mM $Hg(NO_3)_2$ and for a background CV collected in 0.1 $HClO_4$ (black line). **C.** Analyzed Hg deposition and stripping current vs. disk voltage **D.** Cumulative charge associated with Hg deposition and stripping throughout the CV. Light blue arrow indicates start of CV.

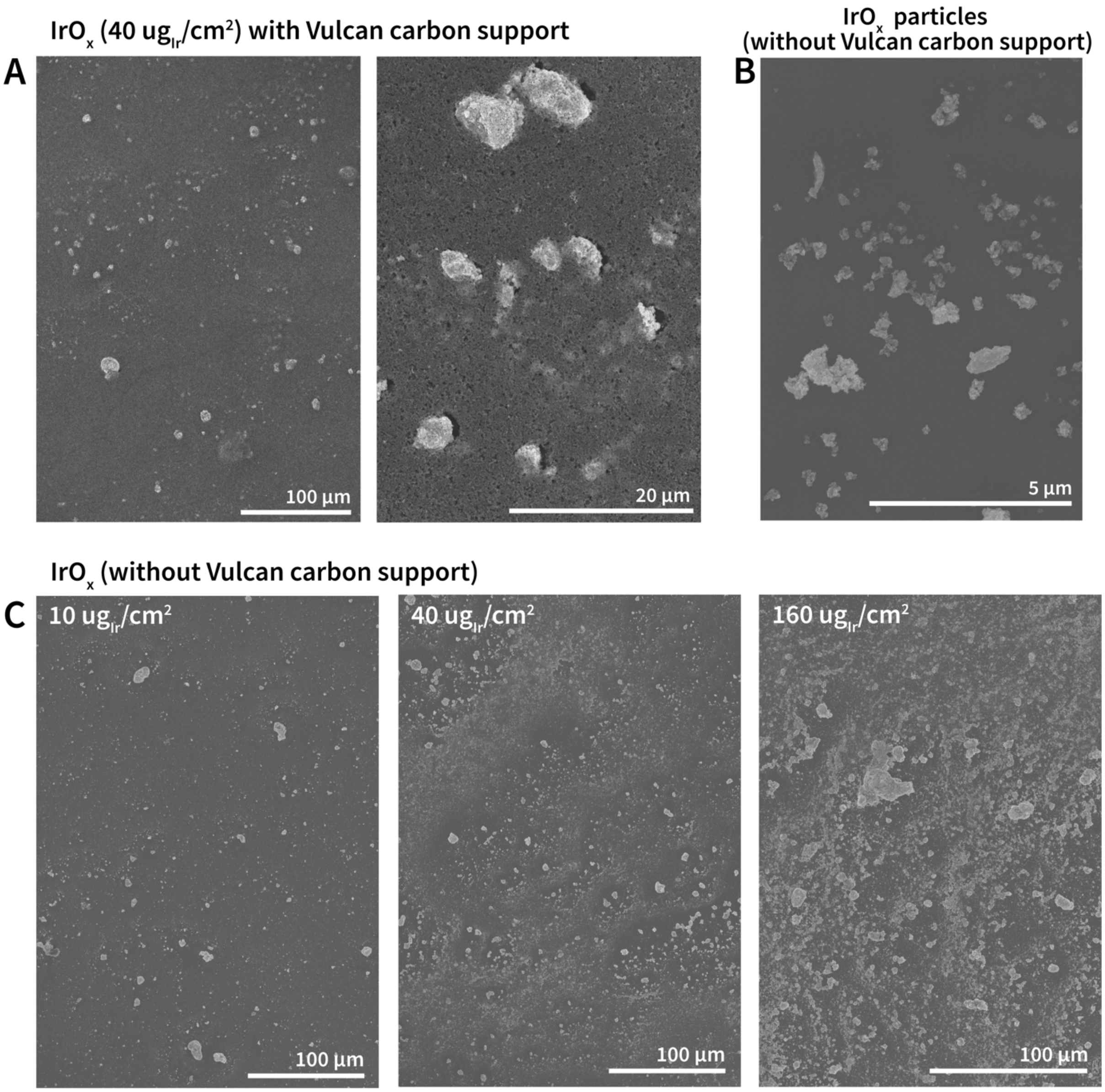

Fig. S16.

SEM images of electrodes with IrO_x. A. An electrode with Vulcan carbon support and 40 $\mu g_{Ir}/cm^2$ IrO_x. **B.** A magnified view of the IrO_x particles on an electrode without glassy carbon, to show characteristic variation in particle size. **C.** Electrodes with various loadings of IrO_x, as studied throughout this work. Images are collected with an accelerating voltage of 10 kV. IrO_x particles appear lighter in contrast to the glassy carbon disk background or Vulcan carbon support.

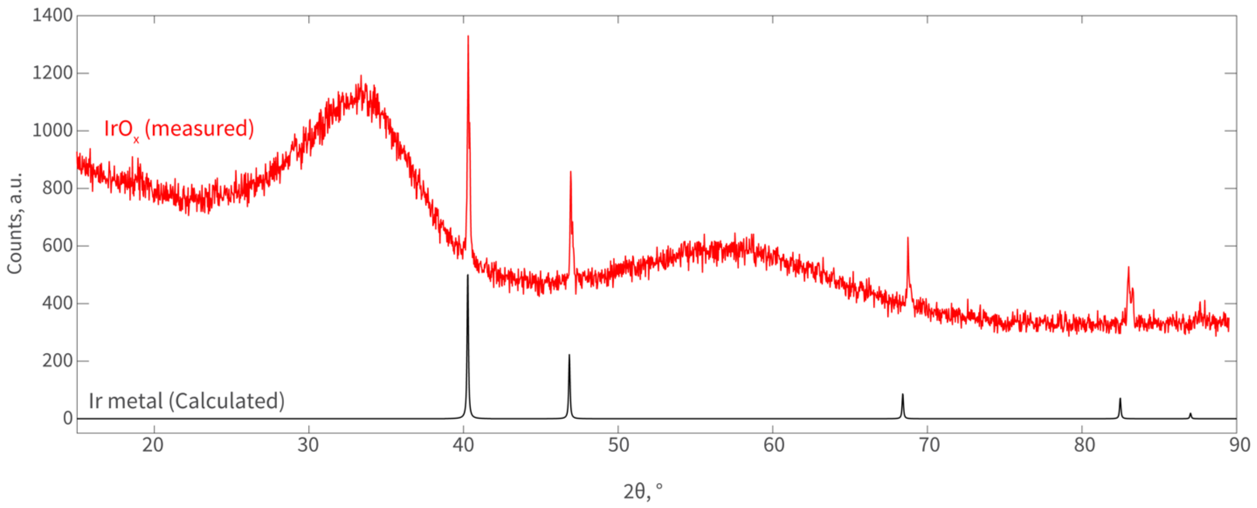

Fig. S17.

Powder X-ray diffraction (PXRD) of commercial amorphous IrO_x powder. A small amount of Ir metal is present in the sample.

Table S1.

Collection efficiency results of clean GC disk and Pt ring using ferrocyanide/ferricyanide system.

**Rotation Rate (rpm)**	**Voltage ($V_{Ag/AgCl}$)**	**$\|I_{disk}\|$ (mA)**	**I_{ring} (mA)**	**N**
1600	-0.1	1.422	0.544	**0.383**
	-0.3	1.465	0.558	**0.381**
	-0.5	1.48	0.562	**0.380**
800	-0.1	1.017	0.3884	**0.382**
	-0.3	1.038	0.3964	**0.382**
	-0.5	1.049	0.3983	**0.380**
400	-0.1	0.724	0.279	**0.385**
	-0.3	0.735	0.283	**0.385**
	-0.5	0.745	0.2845	**0.382**

Table S2.

Collection efficiency results of Pt ring and GC disk with IrO_x (40 $\mu g_{Ir}/cm^2$) and Vulcan carbon using this Hg system.

**Rotation Rate (rpm)**	**Voltage ($V_{Ag/AgCl}$)**	**$\lvert I_{disk} \rvert$ (mA)**	**I_{ring} (mA)**	**N**
1600	-0.1	1.100	0.387	**0.352**
	-0.3	1.283	0.429	**0.334**
	-0.5	1.395	0.445	**0.319**
800	-0.1	0.840	0.303	**0.361**
	-0.3	0.965	0.326	**0.338**
	-0.5	1.030	0.335	**0.325**
400	-0.1	0.643	0.237	**0.369**
	-0.3	0.720	0.244	**0.339**
	-0.5	0.770	0.250	**0.325**

Table S3.

Monolayer occupancy calculation results and analysis for various rotation rates

Rotation rate (rpm)	IrO_x surface area nm^2 (from BET)	# Ir sites (atoms)	# Hg deposited/stripped (atoms)	Hg occupancy per surface Ir site
1600	4.33E+14	2.60E+15	6.67E+14	0.26
800	4.33E+14	2.60E+15	7.30E+14	0.28
400	4.33E+14	2.60E+15	8.24E+14	0.32

Table S4.

Critical data and results for Hg UPD analysis for electrodes with various IrO_x loadings. All Hg UPD results are determined with a rotation rate of 400 rpm.

Ir loading, $\mu g_{Ir}/cm^2$	Ir loading, μg_{Ir}	N	Integration start, C	Integration minimum, C	Integration end, C	Hg deposited, mol	Hg stripped, mol	avg Hg dep/strip, mol	capacitance, mF
10	2.376	0.345	4.20E-06	-8.93E-05	2.57E-06	4.85E-10	4.76E-10	4.80E-10	1.677
20	4.752	0.360	-1.03E-05	-1.38E-4	-7.99E-06	6.62E-10	6.74E-10	6.68E-10	2.275
40	9.504	0.330	1.26E-05	-2.45E-4	1.56E-05	1.33E-09	1.35E-09	1.34E-09	4.728
80	19.008	0.340	5.13E-05	-4.10E-4	3.78E-05	2.39E-09	2.32E-09	2.36E-09	8.958
160	38.016	0.366	7.37E-05	-7.76E-4	-4.29E-05	4.40E-09	3.80E-09	4.10E-09	16.414
40 - no Vulcan support	9.504	0.360	-1.56E-05	-1.76E-4	-2.86E-05	8.32E-10	7.65E-10	7.98E-10	2.932

Table S5.

Monolayer occupancy estimation analysis for electrodes with various IrO_x loadings. All Hg UPD results are determined with a rotation rate of 400 rpm.

Ir loading ($\mu g_{Ir}/cm^2$)	Ir loading, μg_{Ir}	IrO_x surface area nm^2 (from BET)	# Ir sites (atoms)	# Hg deposited/stripped (atoms)	Hg occupancy per surface Ir site
10	2.376	1.08E+13	6.49E+14	2.89E+14	0.45
20	4.752	2.17E+14	1.30E+15	4.02E+14	0.31
40	9.504	4.34E+14	2.60E+15	8.08E+14	0.31
80	19.008	8.67E+14	5.19E+15	1.42E+15	0.27
160	38.016	1.73E+15	1.04E+16	2.47E+15	0.24
40 – no Vulcan support	9.504	4.34E+14	2.60E+15	4.81E+14	0.19
